# Availability and Metabolic Fate of Olive Phenolic Alcohols Hydroxytyrosol and Tyrosol in the Human GI Tract Simulated by the *In Vitro* GIDM–Colon Model

**DOI:** 10.3390/metabo12050391

**Published:** 2022-04-26

**Authors:** Maria Eleni Sakavitsi, Annelies Breynaert, Theodora Nikou, Stef Lauwers, Luc Pieters, Nina Hermans, Maria Halabalaki

**Affiliations:** 1 Division of Pharmacognosy and Natural Products Chemistry, Department of Pharmacy, National and Kapodistrian University of Athens, 15771 Athens, Greece; msakavitsi@pharm.uoa.gr (M.E.S.); th-nikou@pharm.uoa.gr (T.N.); 2 Natural Products & Food Research and Analysis (NatuRA), Department of Pharmaceutical Sciences, University of Antwerp, 2000 Antwerp, Belgium; annelies.breynaert@uantwerpen.be (A.B.); stef.lauwers@uantwerpen.be (S.L.); luc.pieters@uantwerpen.be (L.P.)

**Keywords:** hydroxytyrosol, tyrosol, GIDM–colon, *in vitro* metabolism, LC−Orbitrap MS, gut microbiota, bioavailability, dimerization

## Abstract

Hydroxytyrosol (HTyr) and tyrosol (Tyr) are the most well studied phenolic alcohols of olive oil and olive products demonstrating numerous and significant beneficial health effects. However, their activity in the human organism as food bioactives is strongly associated with their bioavailability and metabolism, while manifested through their metabolites. Nevertheless, there are limited studies investigating their biotransformation and mainly catabolism by gut microflora under a holistic interpretation close to the human organism. Thus, in the present study, the GastroIntestinal Dialysis (GIDM)-colon model, a continuous flow *in vitro* dialysis system mimicking physiological conditions during human gastrointestinal digestion, was used to explore the metabolism of HTyr and Tyr as pure compounds. The GIDM–colon model simulates absorption from the lumen to the mucosa, followed by the colon phase using pooled human fecal suspensions. Samples were collected at different time points and analyzed via LC–Orbitrap MS. An integrated approach combining Multivariate Data Analysis (MVA) and thorough dereplication procedures led to the identification of HTyr and Tyr metabolites in different phases (gastric, small intestine, and colon), yielding also valuable information about metabolites kinetics. To our knowledge, this is the first study reporting full spectrometric data of HTyr and Tyr metabolites along with possible transformation mechanisms in the GI tract.

## 1. Introduction

Hydroxytyrosol (HTyr) or 2–(3,4–dihydroxyphenyl)ethanol and tyrosol (Tyr) or 2–(4–hydroxyphenyl)ethanol are the most well studied phenolic alcohols of olives and olive products, holding numerous beneficial and disease protecting effects [1,2,3]. It is worth noting that HTyr is amongst the most potent natural antioxidants [4]. Both compounds, as well as their analogs, as constituents of olive oil and beyond a certain concentration level, are responsible for a health claim published by EFSA in 2011 supporting their protective effect against LDL oxidation [5]. However, apart from food bioactives, HTyr and Tyr are also endogenous metabolites of the human organism originating from the biosynthetic pathways of dopamine and tyramine, respectively [6,7]. Over the last three decades, *in vitro*, in vivo assays as well as clinical trials have proved their significant bioactivity profile as pure compounds or as part of olive fruit or olive oil [8,9].

As it is well known, their biological and/or pharmacological function is directly associated with their bioavailability [10]. Generally, the biological role of many bioactive compounds in the human organism is attributed to their metabolites [11]. A key element of food bioactives bioavailability determination is the investigation of their metabolism once ingested into the human body [12]. The bioavailability of both HTyr and Tyr has been addressed through ADME(T) studies determining their pharmacokinetic properties [10,11,12,13]. In fact, metabolites of HTyr and Tyr are concentrated in human organs and thus express similar or greater activity than the parent molecules [14,15]. Therefore, acquaintance with the metabolism of bioactive compounds is highly important and fundamental in a drug development process, however, infrequent and rather rare in the area of natural products [16,17].

Metabolism takes place in various tissues, of which the liver and intestine are the main sites for orally administered compounds. Metabolic transformations can be divided mainly into phase I and phase II reactions. Oxidation, hydrolysis, and reduction are common phase I reactions catalyzed by cytochrome P450 (CYP) enzymes, and they are mainly occurring in the stomach and liver. Phase II reactions, also called conjugation reactions, take place mostly in the liver but also in the small intestine and involve the addition of glucuronic, sulfate, glutathionyl, acetyl, methyl moieties on the molecule, catalyzed by respective enzymes [12]. Finally, it is a fact that human gut microbiota contributes significantly to the metabolism of xenobiotics, transforming hundreds of food constituents, chemicals, or pharmaceuticals into metabolites with altered activity, toxicity, and lifetime within the body [18]. However, the information regarding HTyr and Tyr catabolites is limited.

Specifically, for food polyphenols, it has been pointed out that some bioconverted or conjugated forms of polyphenols resulting from phases I, II and colon metabolism are biologically more active than the native forms in which they are present in the diet [19]. Regarding olive polyphenols, it is bibliographically supported that the biological activities ascribed to olive oil consumption are associated in part with its phenolic constituents and their respective metabolites, which are produced in the gastrointestinal (GI) tract [10,20,21].

Furthermore, it is a common conclusion from *in vitro*, in vivo, and human trials that HTyr and Tyr as food bioactives are mainly subjected to Phase I and II metabolism reactions, but they manage to reach the colon and are extensively catabolized by the human gut microbiota. However, their metabolites have not yet been fully described. Around 20 metabolites have been identified so far and some of them have also been characterized as HTyr biomarkers in human biological fluids (plasma or urine). Many of them are common with the in vivo observations, i.e., homovanillic acid (HVA), homovanillic alcohol (HVAlc), 3,4–dihydroxyphenylacetaldehyde (DOPAL), 3,4–dihydroxyphenylacetic acid (DOPAC), 3,4–dihydroxyphenylpropionic acid, protocatechuic acid, which are produced via oxidation of the aliphatic alcohol. Moreover, methylated and generally conjugated metabolites, i.e., sulfates (HTyr–4′–*O*–sulf and HTyr–3′–*O*–sulf), glucuronides (HTyr–4′–*O*–glucuronide and HTyr–3′–*O*–glucuronide), acetates (HTyr–acetate), as well as all the above phase I metabolites in their sulfated and glucoronates forms (HTyr–1–acetate–4′–O–sulf, HVA−sulf, DOPAC−sulf, HVAlc–4′–*O*–glucuronide, etc.), have been suggested [22]. The mercapturate conjugate of HTyr found in rats has not yet been identified in human biological fluids [23].

Regarding Tyr, the literature data so far have revealed that it is exposed to extensive metabolism in the human body, while its bioavailability is deprived in comparison with its metabolites [6,14]. It is important to mention that the absorbed Tyr could be interconverted into HTyr in liver microsomes through the implication of CYP enzymes, therefore, Tyr could be a precursor of HTyr [24,25]. The most abundant metabolites of Tyr are derived from phase–II metabolism, while conjugation reactions lead to Tyr–4′–*O*–glucuronide and Tyr–4′–*O*–sulfate. Moreover, Tyr, lacking the catechol group could not be methylated by COMT [26]. On the other hand, sulfation in the liver appears to be the major metabolic reaction of Tyr, and its metabolites may exert various biological activities in tissues [27]. Similarly to HTyr, it is bibliographically supported that the non–digested Tyr is biotransformed into its catabolites by gut microbiota tο phenylacetic and phenylpropionic acid derivatives. Thus, Tyr shares the last part of gut biotranformation with HTyr [28,29,30,31].

Several *in vitro* methods are routinely used to establish the metabolic profile of a compound, among them the use of microsomes, S–9 fraction, as well as cell–based models, e.g., primary hepatocytes or liver as slices or perfused liver [32]. Complementarily*,* in silico models are used to assist in the selection of the appropriate assay and compounds undergoing further *in vitro* screening [33]. Moreover, they are used to predict metabolism pathways catalyzed by certain enzymes (e.g., P450s) that are involved in the metabolism of compounds [34]. Specifically, for HTyr and Tyr, *in vitro* bioavailability and metabolism assays are restricted to human cell lines (e.g., Caco–2 cells, Hepatoma cells) [26,35]. Even though HTyr and Tyr seem to survive phase I and II metabolism and undergo colonic metabolism by the human gut microbiota [31], there are limited studies referring to *in vitro* protocols for both compounds investigating their metabolic fate in the colon, detecting and/or identifying chemical transformations [29].

Considerably less are the bioavailability and metabolism investigations of HTyr and Tyr in animals [36] and even less in humans [37]. In most cases, similar approaches are followed, including the analyses of biological fluids, mainly plasma and/or excretions, such as feces and urine, trying to quantify the parent molecule and identify metabolites [4,38]. However, there are several constraints since most of the studies are not carried out with pure compounds but as a component of olive oil, which is a very rich and complex matrix containing several HTyr and Tyr derivatives [39]. Moreover, since the phenolic alcohols HTyr and Tyr are involved in endogenous biosynthetic pathways in humans, pharmacokinetic parameters should be carefully evaluated. Endogenously produced metabolic derivatives and their normal levels in human biological fluids should be considered in the design of such studies to reach meaningful conclusions.

Alternative approaches are gradually incorporated to tackle the aforementioned constraints. Specifically, the privilege of an *in vitro* model that mimics the human GI tract could lead to significant new insights into the biotransformation reactions after oral ingestion [40]. Such *in vitro* systems are widely used in drug discovery and development as a part of prioritization of lead compounds [41]. However, their incorporation is limited in food bioactives or natural products. One of the main advantages is the absence of inter–individual variability affecting ADMET parameters such as age, sex, dietary habits, microbiome composition, genetic variation, drug exposure, etc. [42,43]. Moreover, in such *in vitro* systems, the interference of the endogenously produced metabolites is avoided. The *in vitro* GastroIntestinal Dialysis–colon model (GIDM–colon) is such a system, which has been introduced in 2015 by Breynaert and co–workers [40], enriched later with a colonic phase and used in several studies [44,45,46]. For colon phase catabolism, a pooled human fecal suspension is used. It is actually an *in vitro* continuous flow dialysis model including a colonic phase simulating unilateral absorption by passive diffusion from the lumen to the mucosa [47].

Thus, the aim of the present study is to investigate the metabolism of pure HTyr and Tyr using the GIDM–colon system. It could serve as an ideal platform to study HTyr and Tyr metabolism since the few *in vitro* assays that exist mainly focus on the metabolism of phenolic alcohols only in the gut [29]. Furthermore, a UPLC–ESI(–)HRMS and HRMS/MS–based untargeted metabolomic approach was applied for all derived samples to monitor, visualize, and reveal HTyr and Tyr metabolism pathways in the different simulated compartments (stomach, small intestine, and colon). In parallel, a detailed dereplication protocol was applied, taking advantage of the high accuracy and resolution of an Orbitrap analyzer as well as its capability to acquire HRMS/MS spectra for the identification of HTyr and Tyr metabolites. The kinetics of precursor compounds and their gut bacteria interactions were also investigated.

## 2. Results

### 2.1. MVA–PCA Analysis

In the current study, a great challenge was to analyze, monitor, and correlate multiple and diverged LC–MS data obtained from the GIDM–colon model samples. More specifically, a high number of samples was obtained from the different compartments (stomach, small intestine, large intestine), different compounds (HTyr, Tyr), and different time points (0 h, 1 h, 2 h, 4 h, 6 h, and 24 h), while comparison between dialysate and retentate samples in the small intestine has to be considered (Table S1, Figure S1, Supplementary Material). Moreover, in order to reveal meaningful information, the data should be analyzed independently but also in correlation to raise holistic conclusions in a concept of a whole organism approximation. To that end, multivariate data analysis (MVA) and specifically the principal components analysis (PCA) method was used for data projection, classification, and interpretation as well as evaluation of the experimental procedure (GIDM–colon system and UPLC–HRMS analysis). All information regarding time points of sample collection and acquisition set up are properly explained in (Figure S2, Supplementary Material).

**Figure 1:**
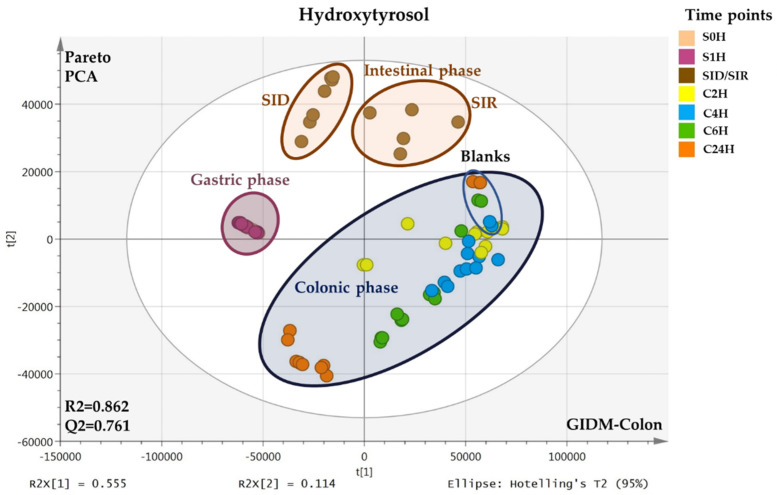

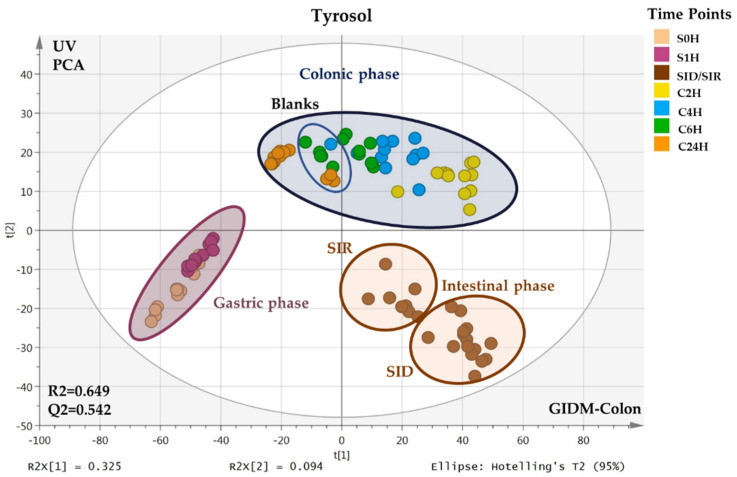
PCA scores plots of HTyr (**upper**) and Tyr (**down**) samples from the GIDM–colon experiment acquired via UPLC–ESI(-)–HRMS. Observations are color coded according to the time points of sample collection: **S0H**, **S1H**, **SID/SIR**, **C2H**, **C4H**, **C6H**, **C24H**. Compartments are highlighted with different colors: Gastric phase (magenta), intestinal phase (brown), and colon phase (dark blue). The blue circled subgroup in the colonic phase corresponds to the colon blank samples (without HTyr or Tyr, respectively).

Thus, all LC–MS data after treatment and processing (Table S2, Supplementary Material) were subjected to PCA, separately for each compound. For the gastric phase (stomach), there are two time points and, therefore, two types of samples immediately at the time of compound addition (**S0H**) and after one hour (**S1H**). For the small intestine, there were two types of samples, one representing the intestinal juice leaving the membrane (**dialysates**, **SID**) and the other, the remaining fraction in the cell (**retentates**, **SIR**) that passes into the colon phase. Both were collected at one time point (1.5 h). In this way, the passive diffusion of intestinal fluids and possible metabolites from the lumen to the mucosa could be simulated. Finally, after the addition of the human colon microbiota (fecal suspension) to the small intestinal retentates, samples were continuously collected at 2 h, 4 h, 6 h, and 24 h (**C2H**, **C4H**, **C6H**, **C24H**). In Figure 1, the PCA scores plots of HTyr (upper) and Tyr (down) GIDM–colon samples are illustrated.

As it is seen in the plots, a clear clustering of the different compartments representing the gastric, intestinal, and colonic metabolism phase is obvious for both compounds. Since the matrix is different in each compartment, it is valid to explore each one separately. Generally, for both HTyr and Tyr, no outliers are observed, while in the colonic phase, the dispersion is high since samples were collected at different time points. This expected scattering indirectly indicates the validity of the generated models but also verifies the catabolism realization in the colonic phase of GIDM–colon model using fecal suspension. It is important to note here that the blank samples were grouped separately from the real samples for both compounds. Interestingly, in the gastric phase, only one group is observed for HTyr, while a clustering trend is observed for Tyr, indicating higher stability in the stomach for the first compared to the latter compound. For both compounds, retentates and dialysates are grouped separately, indicating possibly passive diffusion and therefore, absorption in intestinal phase. 

### 2.2. Identification of HTyr and Tyr Metabolites 

A UPLC–ESI(-)HRMS and HRMS/MS methodology was applied for the detailed dereplication of HTyr and Tyr metabolites. The high resolution and accuracy of the orbitrap analyzer in both full scan and MS/MS levels was the base of the dereplication approach. Chromatographic and spectrometric features, such as RT, suggested elemental composition (EC), ring and double bond equivalence RDBeq, and isotopic patterns together with HRMS/MS spectra. In–house and on–line databases were employed (HRMS and HRMS/MS spectra, Supplementary Material, Figures S5–S29). The mass tolerance for metabolites identification was set to Δm ≤ 5 ppm (Section 4.11) (Table 1 and Table 2).

**Figure 2:**
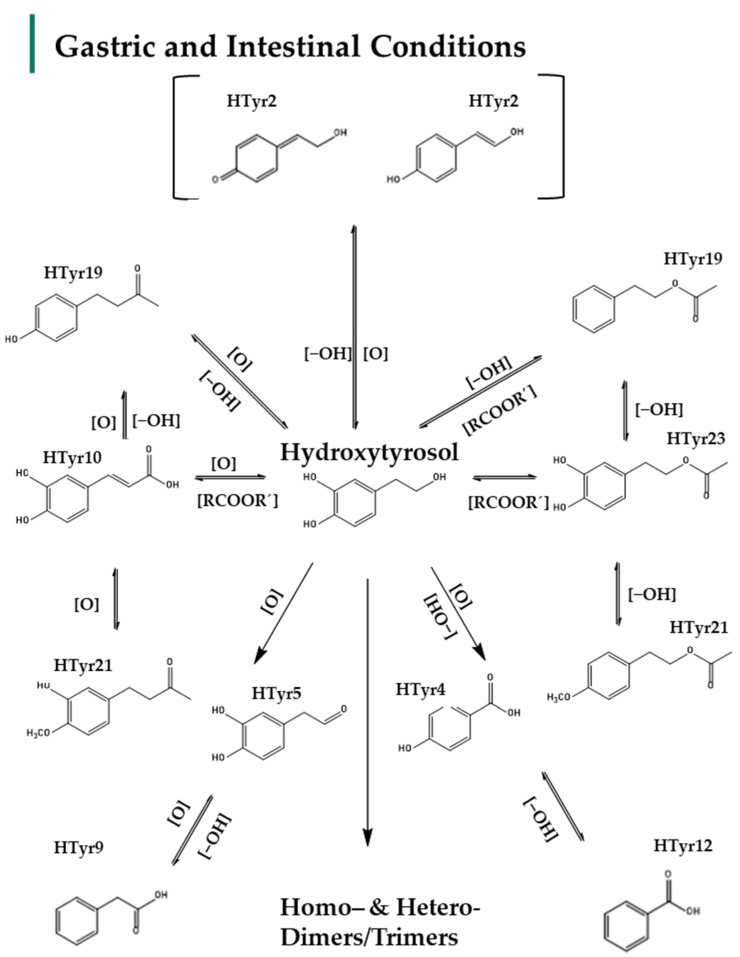
Suggested metabolism pathway of HTyr under gastric (pepsin solution, pH: 2, T = 37 °C) and intestinal conditions (pancreatin, bile solution, pH: 7.5, semi–anaerobic conditions, T = 37 °C). **HTyr2**, **HTyr19**, **HTyr21**, **HTyr23** are not detected in small intestine.

**Figure 3:**
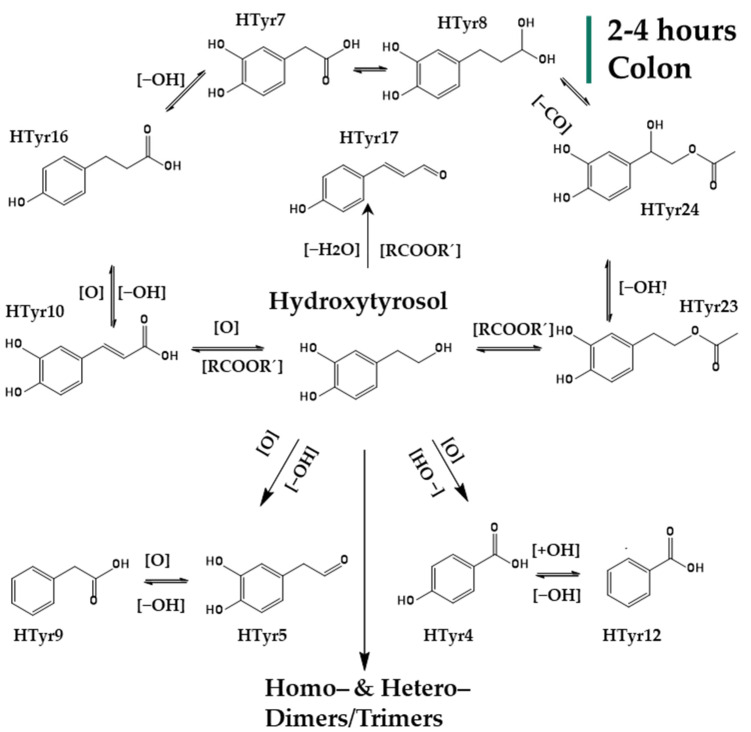
Suggested metabolism pathway of HTyr exposed for 2 to 4 h at colonic conditions (small intestine retentate, fecal slurry, pH: 5.8, anaerobic conditions, T = 37 °C).

**Figure 4:**
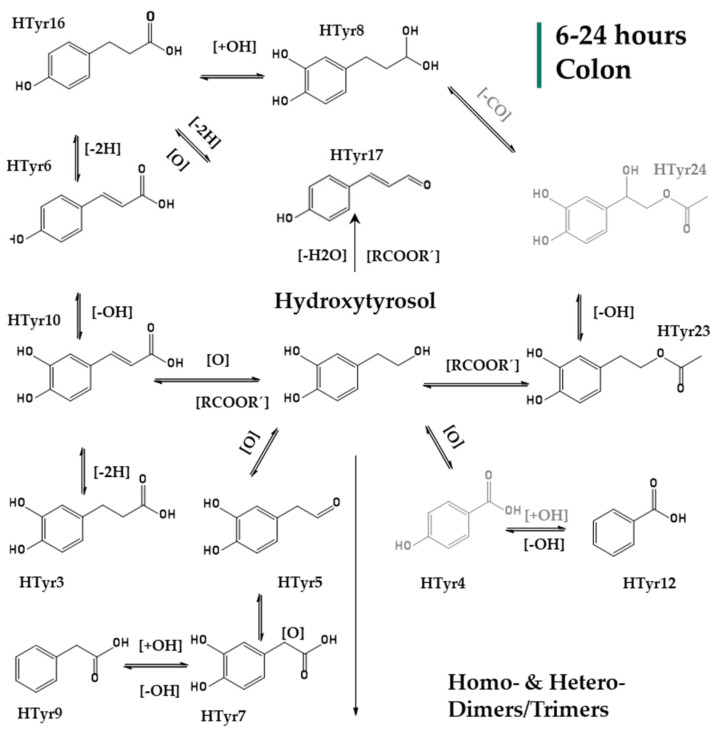
Suggested metabolism pathway of HTyr exposed for 6 to 24 h at colonic conditions (small intestine retentate, fecal slurry, pH: 5.8, anaerobic conditions, T = 37 °C). All metabolites detected (black color) or eliminated (grey color) at the last 18 h of colonic metabolism.

To begin with **HTyr**, the parent molecule was detected immediately after administration to the gastric phase (T = 0 h, S0H and T = 1 h, S1H), in small intestinal retentates and dialysates (SIR/SID) and in all time points in the colonic phase (T = 2 h, 4 h, 6 h, 24 h Colon). However, it was detected in different concentration levels. Overall, 27 HTyr metabolites were putatively identified. Chromatographic and spectrometric information is given in Table 1. Moreover, a schematic representation of the metabolism fate of HTyr in gastric, intestinal, and colon conditions, including its possible metabolites, is given in Figure 2, Figure 3 and Figure 4.

In more detail, the HTyr metabolites detected in all compartments are mostly oxidized forms (e.g., **HTyr3**, **HTyr5**, **HTyr18**), esters (e.g., **HTyr19**, **HTyr23**, **HTyr24**), or methylated (**HTyr21**, **HTyr23**), dehydrogenated (e.g., **HTyr2**), dehydroxylated (e.g., **HTyr19**) derivatives, but also oxidized derivatives with shorter side chain (e.g., **HTyr4**, **HTyr12**) as well as combinations thereof. Due to the structural similarity to Tyr, several metabolites could be structurally considered as Tyr derivatives as well. Amongst these metabolites caffeic, coumaric, and benzoic derivatives have been identified. Interestingly, HTyr dimers (e.g., **HTyr11**, **HTyr13**, **HTyr14**, **HTyr15**, **HTyr26**) that could be divided in homo– and heterodimers as well as to a lesser extent trimers of HTyr (e.g., **HTyr20**, **HTyr22**) have been detected. 

Particularly in the gastric phase, oxidation, dehydroxylation, and esterification reactions mainly occurred. HTyr, as a primary alcohol in a strong acidic environment., is oxidized to aldehydic derivatives, such as DOPAL (**HTyr5**, *m/z* 151.02640, C_8_H_7_O_3_), which are further oxidized to carboxylic acids like dihydrocaffeic acid (**HTyr3**) and caffeic acid (**HTyr10**). Moreover, the carboxylic acids, most probably through *α*–-oxidation, give rise to metabolites with shortened side chains, such as hydroxybenzoic acid (**HTyr4**, *m/z* 137.02514, C_7_H_5_O_3_), phenylacetic acid or PPA (**HTyr9**, *m/z* 135.0454, C_8_H_7_O_2_), and benzoic acid (**HTyr12**, *m/z* 121.0299, C_7_H_5_O_2_) (Table 1). Dehydroxylated forms of HTyr (e.g., **HTyr2**, *m/z* 135.04541, C_8_H_7_O_2_) were detected after 1 h in acidic environment, though they were eliminated after being subjected to the alkaline small intestine conditions. Moreover, it seems that the simultaneous presence of carboxylic acids and alcohols in the acidic medium leads to esterification products like HTyr acetate (**HTyr23**, *m/z* 195.06632, C_10_H_11_O_4_). Moreover, the metabolites **HTyr19** (*m/z* 163.0767, C_10_H_11_O_12_) and methylated **Htyr21** (*m/z* 193.0871, C_11_H_13_O_3_) could support either products of esterification or products of oxidation resulting in phenylethyl acetate/or 4–(4–hydroxyphenyl)–-2–oxobutane for **HTyr18** and 4–methoxyphenethyl acetate or 4–(3–hydroxy–4–methoxy–phenyl)–butan–2–one for **Htyr19**, respectively (Table 1). Finally, as mentioned already, the acidic conditions favor the formation of C–C dimers, which seem to be composed of two HTyr units (homodimers) or one HTyr and one Tyr unit (heterodimers). The most characteristic and abundant is the heterodimer **HTyr26** *(m/z* 289.10779, C_16_H_17_O_5_)**.** In addition to **HTyr26**, other less abundant dimers were detected involving dimerization of two HTyr units, such as **HTyr15** (*m/z* 305.1028, C_16_H_17_O_6_) or the heterodimer **HTyr25** (*m/z* 303.0870, C_16_H_15_O_6_). It is notable that enzymatic esterification in water is possible at a slightly alkaline pH but is rapid in acidic conditions [48].

The study of small intestine samples that represent the fraction that passed from lumen to mucosa through passive diffusion (dialysate), as well as retentate samples that correspond to the fraction that later sustained for colon metabolism, resulted in several metabolites. HTyr was hardly detected in both fractions as well as its main dimer **HTyr26**. The same fate had all the esterified derivatives and the oxidized derivatives of HTyr. However, caffeic acid (**HTyr10**) is present in both fractions along with **Htyr4**, **HTyr5**, **HTyr9,** and **HTyr12** (Table 1). Moreover, it seems that in slightly alkaline conditions, multiple hydroxylation reactions seem to occur, giving rise to hydroxylated homo–and heterodimers such as **HTyr11** (*m/z* 353.0872, C_16_H_17_O_9_) and **HTyr14** (*m/z* 335.0766, C_16_H_15_O_8_). Interestingly, the formation of trimers also seems to occur, which is not detected in the strongly acidic environment of the gastric phase. Specifically, three trimers, i.e., **HTyr20** (*m/z* 459.1291, C_23_H_23_O_10_), **HTyr22** (*m/z* 443.1341, C_23_H_23_O_9_), and **HTyr27** (*m/z* 441.1185, C_23_H_21_O_9_), have been detected. 

After entering the colon phase, an explosion of metabolites was observed as a function of time supporting an obviously increased metabolic activity induced by colon conditions. Interestingly, **HTyr** and its dimer **HTyr26** are detected again together with hydroxytyrosol acetate (**HTyr23**) and DOPAL (**HTyr5**). Caffeic acid (**HTyr10**), which was the most abundant metabolite during the intestinal phase, is still present in the first two hours of the colon phase, but with an elimination trend through time. As it is shown in Figure 3, after 2 h of exposure to colonic conditions, all the key metabolites are quite stable at least for 4 h (T = 4 h). Moreover, HTyr is still detected after 24 h incubation as well as its dimer, while caffeic acid seems to be eliminated after 6 h (Figure 4). Specifically, after 4 h, at colon conditions, hydroxyphenylpropionic acid (**HTyr16**, *m/z* 165.05594, C_9_H_9_O_3_) was detected along with its dehydrated form **HTyr17** (*m/z* 147.0454, C_9_H_7_O_2_). Moreover, DOPAC (**HTyr7**, *m/z* 167.0428, C_8_H_7_O_4_) and 4–(1,3–dihydroxypropyl) benzene−1,2−diol (**HTyr8**, *m/z* 183.0665, C_9_H_11_O_4_) were detected together with HTyr acetate (**HTyr24**, *m/z* 211.0611, C_10_H_11_O_5_). Finally, a dehydroxylated form of **HTyr27** appeared, namely, **HTyr13** (*m/z* 273.0766, C_16_H_17_O_4_). Moving from 4 to 24 h of colonic metabolism, dihydrocaffeic acid (**HTyr3**, *m/z* 181.05087, C_9_H_9_O_4_), a dimer of HTyr (**HTyr13**, *m/z* 273.0766, C_16_H_17_O_4_), coumaric acid (**HTyr6**, *m/z* 163.04046, C_9_H_7_O_3_), and the phenylacetaldehyde (**HTyr18**, *m/z* 119.0505, C_8_H_7_O_0_) were detected. 

**Figure 5:**
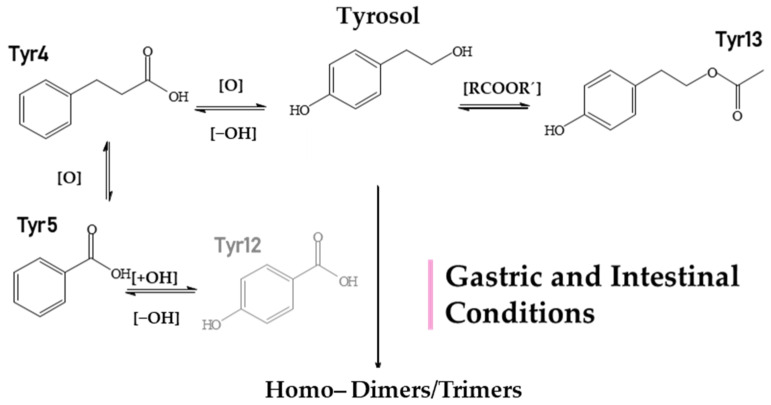
Suggested metabolism pathway of Tyr under gastric (pepsin solution, pH: 2, T = 37 °C) and intestinal conditions (pancreatin, bile solution, pH: 7.5, semi−anaerobic conditions, T = 37 °C). **HTyr12** is not detected in the small intestine.

**Figure 6:**
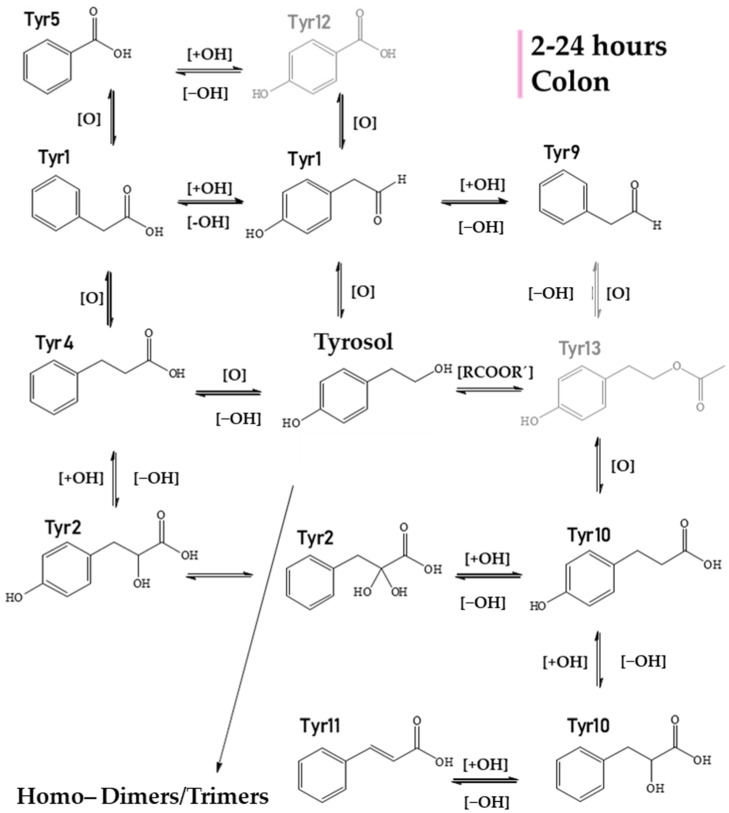
Suggested metabolism pathway of **Tyr** exposed for 2 to 24 h at colonic conditions (small intestine retentate, fecal slurry, pH: 5.8, anaerobic conditions, T = 37 °C). All metabolites detected (black color) or eliminated (grey color) during 24 h of colonic metabolism.

**Figure 7:**
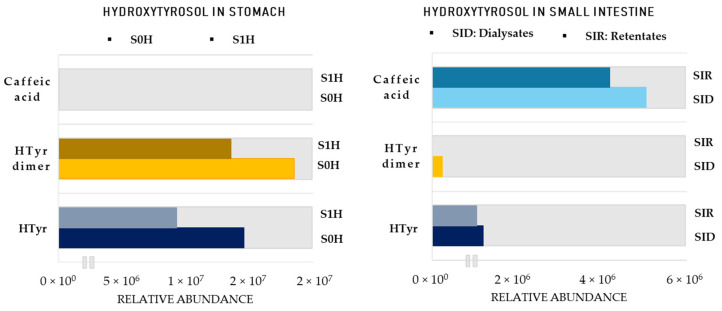
Area of HTyr metabolites in the stomach and small intestine. **S0H**: stomach Τ = 0 h; **S1H**: stomach Τ = 1 h; **SIR**: small intestine retentates, T = 1.5 h; **SID**: small intestine dialysates, T = 1.5 h. **HTyr dimer**: **Htyr26**.

A similar workflow was followed for the identification of Tyr metabolites through the GIDM−colon model. It is important to note here that Tyr was subjected in the GIDM−colon model as a standard compound at four different concentrations (0.01 ug/mL, 0.1 ug/mL, 10 ug/mL, 100 ug/mL) since it served as a model compound but also due to the fact that Tyr cannot be easily detected under ESI(-) conditions. Preliminary analysis of the derived samples took place, which allowed us to investigate the behavior of Tyr (in solvent and in matrix) and determine the appropriate limit of detection (Figure S21, Supplementary Material). Unfortunately, Tyr ionization was quite limited even at the highest concentration (100 μg/mL), which is in agreement with the existing literature [49]. However, Tyr could be detected as its on–source dimer [2M–H]^−^ (*m/z* 273.1137, C_16_H_17_O_4_, RDBeq. 8.5) and therefore, it was detected in this form in the different samples. Finally, 13 metabolites of Tyr were putatively identified, and their suggested structures are presented in Figure 5, Figure 6 and Figure 7.

Starting with the gastric phase (Figure 5, Table 2), **Tyr** undergoes similar transformations as HTyr in a strongly acidic environment. Phenylpropionic acid (**Tyr4**, *m/z* 149.0248, C_8_H_5_O_3_) is one of the most characteristic oxidized and dehydroxylated derivatives. Subsequently, through *α*−and *β*−oxidation reactions, benzoic acid (**Tyr5**, *m/z* 121.0295, C_7_H_5_O_3_) and 4–hydroxybenzoic acid (**Tyr12**, *m/z* 137.0293, C_7_H_5_O_3_) are formed. Following the same fate as HTyr, the simultaneous presence of carboxylic acids and alcohols in an acidic medium lead to esterification products like tyrosol acetate (**Tyr13**, *m/z* 179.0717, C_10_H_11_O_3_). 

The study of the small intestine samples resulted in Tyr metabolites described in the stomach phase, except for 4−hydroxybenzoic acid (**Tyr12**), which was not detected. Furthermore, as for Tyr, a dimer and a trimer were detected (**Tyr6**, **Tyr8**) with more pronounced trimer **Tyr6** (*m/z* 409.1657, C_24_H_25_O_6_). It is important to note here the polymerized metabolites that were observed contain only Tyr units (homodimers and homotrimers). It is worth noting that much fewer Tyr metabolites were detected in both phases compared to HTyr.

Entering the colon phase (Figure 6, Table 2), an increase in the number of metabolites was observed due to catabolic reactions by microflora. Particularly, during the first 2 h of the colon phase, only phenylpropionic acid (**Tyr4**), hydroxylated tyrosol dimer (**Tyr8**), hydroxybenzoic acid (**Tyr12**), and tyrosol acetate (**Tyr13**) could survive. Tyrosol trimer (**Tyr6**) was completely eliminated, but hydroxylated tyrosol dimer (**Tyr8**, *m/z* 289.1082, C_16_H_17_O_5_) was still present (Table 2). During the next 2 h of colonic metabolism (T = 4 h), the metabolic profile of Tyr metabolites started to flourish with the reappearance of benzoic acid (**Tyr5**), Tyr (**Tyr7**) and the addition of two chemical entities, namely, hydroxyphenylpropionic acid (**Tyr10**, *m/z* 165.05594, C_9_H_9_O_3_) and cinnamic acid (**Tyr11**, *m/z* 147.0456, C_9_H_7_O_2_). On the other hand, 4−hydroxybenzoic acid (**Tyr12**) and tyrosol acetate (**Tyr13**) were totally eliminated after 4 h of colonic metabolism. Similarly to HTyr, moving from 4 to 6 h of catabolism (Figure 6, Table 2), a downward trend of all key metabolites of Tyr was observed, whereas from 6 h to 24 h, their relative abundance was quite stable. More specifically, after 6 h of colonic metabolism two more metabolites, dihydroxyphenylpropionic acid (**Tyr2**, *m/z* 181.0509, C_9_H_7_O_3_), and phenylacetaldehyde (**Tyr9**, *m/z* 119.0505, C_8_H_7_O), were detected while after 24 h of colonic metabolism, phenylacetic acid (**Tyr1**, *m/z* 135.0455, C_8_H_7_O_2_) and coumaric acid (**Tyr3**, *m/z* 163.0404, C_9_H_7_O_3_) were detected.

## 3. Discussion

In the current study, the continuous dialysis model with a colon phase simulating gastric, intestinal, and gut conditions (GIDM−colon) of the human GI track was employed to explore the availability and metabolic fate of olive phenolic alcohols HTyr and Tyr. As already mentioned, the GIDM−colon model could serve as an ideal platform since it is a good approximation of metabolism of orally administrated compounds, such as food bioactives. The interindividual variability is avoided, as well as the interference of endogenously produced metabolites, such as in the case of HTyr and Tyr, which are also produced through dopamine and tyramine pathways, respectively [6,7,40]. Furthermore, GIDM−colon model enables the investigation of the compounds of interest in pure form and not as a component of a more complex mixture, such as in the majority of the in vivo and human studies, which use olive oil or olive oil polyphenols extracts to study HTyr and Tyr metabolism [37,50].

Nevertheless, one of the most challenging and demanding parts of the study was to treat and handle numerous divergent samples and their corresponding LC−HRMS data in order to extract meaningful information (Figures S1 and S2, Supplementary Material). A general LC−MS methodology was developed and incorporated for both compounds (Section 4.9). To that end, the first step was the use of untargeted LC−MS−based metabolite profiling approach and MVA methods to visualize, compare, and classify our data in a more holistic and unbiased way. Specifically, the unsupervised descriptive PCA method was employed separately for the dataset of each compound. Indeed, a clear classification between the three compartments (stomach, small intestine, colon) was revealed for both compounds when exposed to GIDM−colon system. This result could be expected due to different matrix at gastric, intestinal, and colonic conditions, however, it verifies the good performance of the system. For the same reason, the most valid and important observations to discuss are within each of all the three compartments (Figure 1).

Specifically, starting with the gastric phase of HTyr, no sub−clustering is observed, which probably indicates the stability of HTyr under digestate conditions, which is in accordance with the literature [51]. In contrast, sub−clustering is observed in Tyr, indicating a digestive instability or at least less stability to HTyr, probably due to the absence of the catechol group [52]. Subsequently, the transition from the stomach to small intestine is accompanied by a significant pH change (pH = 2 to pH = 7.5) and the simulation of passive diffusion from lumen to mucosa, using the dialysis cells equipped with membranes (Section 4.2). For both compounds, a sub−clustering between retentates and dialysates is observed, indicating passive diffusion, and therefore, absorption in the small intestine. This is in accordance with previous studies reporting that HTyr absorption mainly occurs by passive transport in the small bowel and the colon at a rate from 75% up to 100% [8,38]. As for the colonic phase, clear clustering according to the time points is observed in both PCA scores plots, which is the first evidence of metabolic activity of the gut microbiota, while extensive catabolism occurs after 24 h (Figure 1).

**Figure 8:**
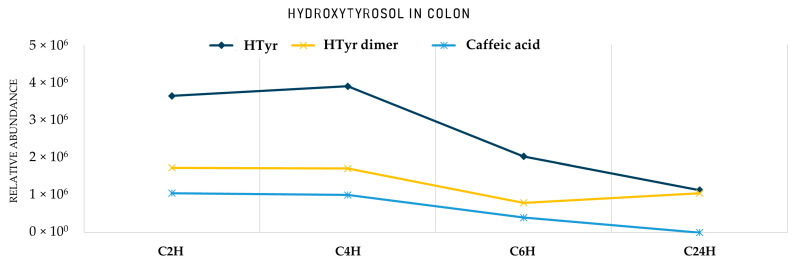
Area of HTyr metabolites in the colon. **C2H**: Colon Τ = 2 h; **C4H**: Colon Τ = 4 h; **C6H**: Colon Τ = 6 h; **C24H**: Colon Τ = 24 h. **HTyr dimer**: **Htyr26**.

After these first useful observations, all data were examined using a thorough dereplication protocol resulting in 27 putatively identified metabolites of HTyr and 13 of Tyr, in all three metabolism phases. More than double HTyr metabolites were detected, compared to Tyr as a result of the more reactive nature of HTyr (Figure S3, Supplementary Material). Due to the presence of the catechol group, an equilibrium of quinone methide with high electrophilic reactivity and its tautomer *o*–quinone could be easily sustained, facilitating oxidation and further biotransformation reactions, giving, therefore, rise to several metabolization products [51]. To that end, and in order to give a relative quantitative dimension and explore kinetics in the colon, we focused on key metabolites in each metabolization phase. Specifically, major metabolites were monitored in the different compartments based on their respective peak area (Figure 7 and Figure 8). Starting with the stomach, HTyr (**HTyr1**) is detected in comparable levels signifying its stability in acidic conditions. Interestingly, its heterodimer **HTyr28** shows similar abundance as well, while caffeic acid is not detected even after 1 h in an acidic environment.

In contrast to the stomach, in the small intestine, HTyr and its dimer are detected at considerably low levels, while caffeic acid is detected in high amounts, indicating transformation reactions. It seems that HTyr, as long as it is present in the semi−aerobic small intestine conditions, is rapidly eliminated, giving rise to caffeic acid (HTyr10), at least to a certain extent (Figure 8). Regarding the comparison between retentates (SIR) and dialysates (SID), caffeic acid seems to be abundant in equal amounts in both matrixes, indicating that it could also be absorbed from the small intestinal compartment by passive diffusion. 

Finally, the transition from the small intestine to strictly anaerobic colon conditions and slightly acidic pH gave noteworthy rise to the relative abundance of the precursor HTyr as well as its dimer (**HTyr26**). Specifically, moving forward at the 2 h of gut metabolism, an increase in the total area of HTyr is observed, which is gradually decreased through time. However, both could be detected even after 24 h of catabolism. On the other hand, caffeic acid is abundant in low levels after 6 h and it is not detectable anymore after 24 h. An interesting observation is that both HTyr and its dimer are detected in very low abundance in the small intestine compared to the gastric and colonic phase. A reasonable hypothesis could be that both HTyr and its dimer are bound to bile acids (Bas) in the small intestine and as soon as microbiota start to metabolize them in the colonic phase, Htyr and its dimer are released again in free form. The amphiphilic nature of Bas bearing a hydrophilic and a lipophilic surface might possibly act as a binding or trapping substrate of Htyr, functioning as carriers of HTyr. Such Bas−drug [53] interactions have been previously reported for numerous drugs, such as BAs sequestrants and microlides [54]. The implication of BAs may play a critical role in the bioaccessibility and bioavailability of HTyr.

On the other hand, and regarding Tyr, it seems that no selective hydroxylation at position 3 (C−3) of the phenyl ring of Tyr was observed since HTyr was not detected and, therefore, no caffeic acid derivatives can be formed. Additionally, it seems that the absence of the 3−OH has a great influence on the metabolism profile of Tyr. In comparison to HTyr, only about half metabolites were identified. This minimal scenery during the metabolism of Tyr is either a result of poor ionization of the existing metabolites and/or the structural difference with HTyr, which makes Tyr less prone to reactions.

**Figure 9:**
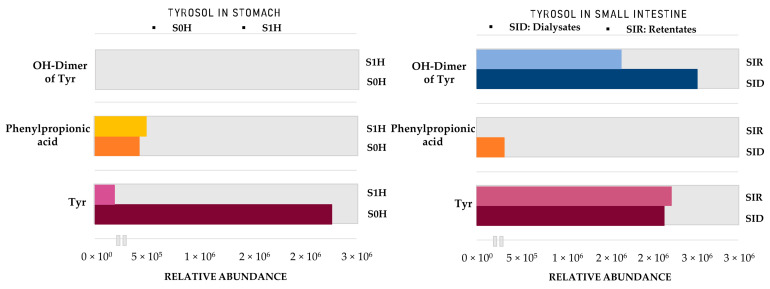
Area of Tyr metabolites in Stomach and Small Intestine. **S0H**: Stomach Τ = 0 h; **S1H**: Stomach Τ = 1 h; **SIR**: Small intestine retentates, T = 1.5 h; **SID**: Small intestine dialysates, T = 1.5 h. **Tyr**: Tyrosol; **OH**−**Dimer of Tyr**: Hydroxylated dimer of Tyr (**Tyr8**).

**Figure 10:**
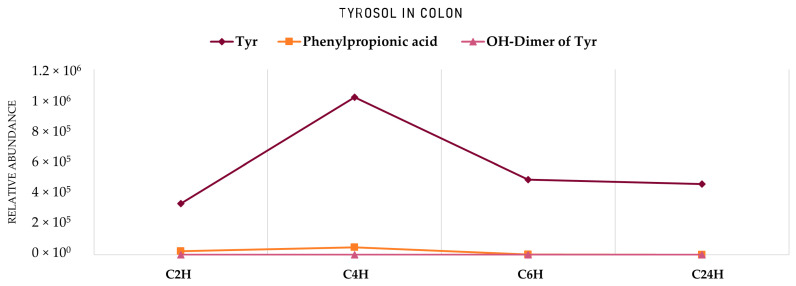
Area of HTyr metabolites in the colon. **C2H**: Colon Τ = 2 h; **C4H**: Colon Τ = 4 h; **C6H**: Colon Τ = 6 h; **C24H**: Colon Τ = 24 h. **Tyr**: Tyrosol; **OH**−**Dimer of Tyr**: Hydroxylated dimer of Tyr (**Tyr8**).

As shown in Figure 9, Tyr levels significantly decrease after 1 h in acidic conditions, which was also revealed from PCA plots. On the other hand, phenylpropionic acid seems to be immediately formed as soon as Tyr is introduced into the acidic environment. In contrast to HTyr even if dimers have been detected in the gastric phase, its characteristic hydroxylated homodimer (**Tyr8**, *m/z* 289.1082, C_16_H_17_O_5_), is mainly formed only after passing to intestinal conditions (pH = 7.5). In the small intestine, both Tyr and its dimer seem to pass from lumen to mucosa in comparable amounts. In contrast to HTyr, they are both detected in respectful amounts, indicating no interaction with BAs. As far as the colonic phase is concerned, it seems that from those three compounds only Tyr significantly survives catabolism after 24 h (Figure 10). Of course, other compounds, such as benzoic and cinnamic acid, are also detected (Table 2).

Overall, 27 metabolites of HTyr (Table 1) and 13 metabolites of Tyr (Table 2) were putatively identified, and an effort to give possible chemical structures was made for HTyr (Figure 2, Figure 3 and Figure 4) and for Tyr (Figure 5 and Figure 6). They concern mostly hydroxycinnamic acid derivatives, esterified derivatives, phenylacetic acid and benzoic acid derivatives and also dimers and trimers. Many of these metabolites have been suggested before in the literature. So far, approximately 20 metabolites of phase I and phase II metabolic reactions have been proposed for HTyr and Tyr. Briefly, methylated forms of HTyr [55], aldehydes, and acids formed via oxidation of the aliphatic alcohol [36], sulfates and glucuronides [26,56,57] acetylated [58,59], and N−acetylcysteine derivatives [23], have been reported. 

**Figure 11:**
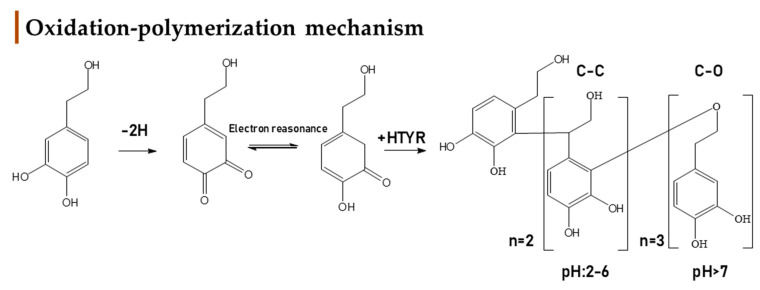
Mechanistic approach of HTyr’s oxidation and dimerization under conditions described in bibliography [61,67,68].

In the present study, during GIDM-colon metabolism, HTyr metabolites detected were products of oxidation, hydroxylation, dehydroxylation, esterification, reduction of *pi*−bonds of caffeic acid, and polymerization reactions. As such, Tyr metabolites were obtained through oxidation, dehydroxylation, esterification, and polymerization reactions. Both compounds and their metabolites suffered catabolism by colonic microflora. It is important to note that active catabolism was observed when HTyr was incubated with human fecal slurry. According to the literature, colonic metabolism of HTyr yields mostly to phenylacetic and phenylpropionic acid derivatives. Indeed, our study agrees with the previously reported metabolites. Phenylacetic acid was the main end catabolite through the fermentation in all the samples, reaching the 24 h presence in the colon [29,30,31]. As expected, sulfoconjugated metabolites and glucuronide conjugates have not been detected due to the absence of the relevant enzymes. Apart from the possible interactions of HTyr metabolites with BAs and matrix proteins, no further conjugation was observed. According to our results, the same conclusion can be drawn for Tyr as well. Finally, a significant observation during metabolism of HTyr and Tyr through GIDM–-colon is autooxidation products, including homo– or hetero–dimers and trimers of parent compounds that are firstly associated as HTyr and to a lesser extent as Tyr metabolites (Figure 11). 

Oxidation and polymerization of *ortho*−diphenols have attracted scientific interest in the past sporadically in order to understand the mechanism behind antioxidants. Knowledge of the oxidative chemistry of HTyr is of central relevance for understanding the fate of this diphenolic compound during its antioxidant activity in vivo and for describing the chemical processes underlying quality deterioration of olive oil [60]. The chemistry behind it involves the formation of the corresponding and highly reactive *o*−quinones, which could take place even in the absence of enzymes, especially when the medium is alkaline. As oxidants, *o*−quinones oxidize other products with lower redox potentials, for instance, other phenolic compounds, and are reduced to the original phenol. *O*−quinone may condense with the corresponding hydroquinone, either through a Michael addition type reaction, or through a mechanism involving semiquinone radical intermediates and polymerization [61]. Catechols are mostly dimerized at pH < 7, but drastically increase their polymerization degree at pH > 7 due to the thermodynamic stability of semiquinone radical, which becomes abundant enough to afford trimers (Figure 11) [62,63,64].

In the case of Tyr, the literature data that were found regarding polymerization are very limited. Based on the bibliography, oxidation of Tyr could happen only enzymatically, leading to HTyr formation which can potentially form other metabolic and polymeric products [65,66]. In the present study, the non−catecholic Tyr scaffold, in combination probably with the absence of hydroxylation reaction during GIDM −colon, significantly restricted the mechanism of Tyr polymerization.

Another crucial matter is the type of polymerization, formation of C−C or C−O bonds, during the autooxidation reaction. The C−C linkage in the polymer is mostly produced at an acidic pH, whereas the C−O linkage dominates at an alkaline pH [67]. Remarkably, in the case of *o*−diphenols with unconjugated chains, such as HTyr and dihydrocaffeic acid, dimerization can occur with incorporation of a water molecule. Hence, it can be proposed that *o*−quinones derived from HTyr and dihydrocaffeic acid, or more probably tautomeric *p*−quinone methides, undergo water addition before oxidative coupling with a second *o*−diphenol molecule (Figure 11) [68]. It is supported that two types of caffeic acid dimers can be proposed, according to their fragmentation, one having unbreakable C− C linkages (‘C−C dimers’, e.g., biphenyl type), and the other having breakable C−O linkages (‘C−O dimers’, e.g., biarylether type). 

Oxidation and polymerization of HTyr are in accordance with the metabolic profile of HTyr, convoyed with LC–MS/MS data (Figures S4–S29, Supplementary material), deriving from *in vitro* metabolism with GIDM–-colon. In our study, dimerism of HTyr was firstly noticed during gastric conditions at pH = 2 where esterified, oxidized, and dimerized derivatives were detected. The most abundant dimer was **HTyr26** (MS/MS: 135, 153), together with **HTyr25** (MS/MS: 167, 123) and **HTyr15** (MS/MS: 275,179,161). All dimers were a C–C form, as expected due to the acidic environment. Indeed, in addition to the fragmentation pattern common to both types of dimers (decarboxylation), the C–O dimers give monomeric fragments, whereas the C–C dimers do not. Moreover, the C–O dimers display one less OH group and so are less polar than the C–C dimers and are consistently eluted later on the reverse phase column. Even if dimers of HTyr have been proposed before, in none of the previous studies, the human gastric conditions (pH 2, pepsin solution, T = 37 °C, 1 h shacking bath, Materials and Methods) [58,60,64,68] have been simulated or none of the research aiming the study of HTyr metabolism *in vitro* was focused on gastric conditions in such detail since HTyr, like other dietary phenols, is mainly catabolized from colonic microbiota [29]. Furthermore, no further degree of polymerization was noticed.

Moving towards the small intestine an abrupt decrease in HTyr was noticed together with its main dimer **HTyr28** (Figure 7), while only the hydroxylated dimers **HTyr11** (MS/MS: 235,265,247) and **HTyr14** were detected and trimers of HTyr appeared instead. Specifically, the increase of the pH to 7.5 let the degree of polymerization increase and formed C–O bonds between the HTyr moieties. **HTyr20**, **HTyr22,** and **HTyr27** were the detected metabolites of HTyr during the small intestine phase. Thus, a rational hypothesis could be that the almost complete absence of HTyr and its dimer is due to the polymerization reaction or/and conjugation reactions with BAs or proteins of the luminal medium.

Finally, under the colon conditions, a completely different metabolic profile is arising with trimers being eliminated completely and dimers reappearing. Characteristic metabolites present in the colon phase were **HTyr13**, **HTyr25,** and **HTyr26**. The latest two forms were in common with the gastric phase of metabolism. Htyr and its main dimer are detected again, strengthening the hypothesis that BAs and/or trimers of HTyr could serve as carriers of HTyr to the colon and could be considered as prodrugs. 

Regarding Tyr, only a homo–dimer **Tyr8** (MS/MS: 271, 121) and a homo–trimer **Tyr6** (MS/MS: 273) were firstly detected, during intestinal and slightly alkaline conditions, while **Tyr8** was also available during colonic catabolism. Based on the bibliography, under alkaline conditions enzymatic oxidation of Tyr led to HTyr, an *o*–quinone that subsequently generates the detected di–/trimers. Unfortunately, such intermediate *o*–quinone was not detected in Tyr dataset to support this mechanistic approach [65,66].

## 4. Materials and Methods

### 4.1. Chemicals and Materials

Hydroxytyrosol (2– (3,4–dihydroxyphenyl) and chlorogenic acid (5–O–caffeoylquinic acid, 95%) used as quality control standard of fermentation metabolites, and ethanol, ≥98.0%), methanol Chromasolve^®^ HPLC grade, pepsin (P–7000, from porcine stomach mucosa, 800–2500 U/mg protein), bile salt (B–8631, porcine), pancreatin (76,190, from hog pancreas, 149 USP U/mg amylase), sodium dihydrogen phosphate anhydrous (NaH_2_PO_4_), disodium phosphate dihydrate (Na_2_HPO_4_ × 2H_2_O), sodium thioglycolate broth, glycerol suitable for culture was used in all experiments, were purchased from Sigma–Aldrich (St. Louis, MO, USA). Deionised water (milliQ, Millipore). Hydrochloric acid (HCl, 32 wt.% for analysis), *ortho*–phosphoric acid (H_3_PO_4_, 85%), sodium bicarbonate (NaHCO_3_), sodium hydroxide pellets (NaOH), and ethanol were obtained from Merck (Darmstadt, Germany). All chemicals and reagents were of analytical grade. Acetonitrile and methanol (ACN, MeOH, ≥99.9%, LC–MS grade) were obtained from Fisher Scientific (Hampton, NH, USA).

### 4.2. Equipment

Dialysis tubing with a molecular weight cut–off of 12 to 14 kDa (Visking size 6 Inf Dia 27/32–21.5 mm: 30 M) was purchased from Medicell Ltd. Membranes were stored at 4 °C in a 20% (*v:v*) EtOH solution. Before use, dialysis tubings were rinsed three times with deionized water (10 min each time). Stirred ultrafiltration cells (model 8200, 200 mL, 63.5 mm diameter), the associated controller (controller MF2 and a reservoir RC800), and the ultrafiltration discs (Ultracel molecular weight cutoff 1000, 63.5 mm diameter) were purchased from Millipore. The disposable paper collection devices, Protocult^®^, were from Ability Building Centre, the receiver VR faeces D41 × 57 mm, Bagpage^®^ 400 mL sterile filter bags, and the Stomacher Lab Blender were purchased from VWR. The Globe Box (Jacomex Globe Box T3) to create the anaerobic environment was purchased from TCPS.

### 4.3. Preparation of Digestive Juices

One gram pepsin was dissolved in 100 mL of 0.01 mol/L HCl (6220 FIP –U/ml). Pancreatin–bile mixture was prepared by dissolving 0.4% (*w:v*) pancreatin and 0.76% (*w:v*) bile in 0.1 mol/L NaHCO_3_ [32,000 FIP–U lipase, 143,600 FIP–U amylase, 6400 FIP–U protease and 17.9 mmol bile/L (for lipase: 1 FIP–unit = 1 USP unit [United States Pharmacopoeia]; for amylase: 1 FIP–unit = 4.15 USP units; for protease: 1 FIP–unit = 62.5 USP units). Pancreatin is mainly obtained from the pancreas of pigs, as the human digestive juice is comparable to that of pigs.

### 4.4. Preparation of the Fecal Slurry

One human fecal donor (*n* = 1) was selected who met the following inclusion criteria: Female, 25–45 years of age, non–pregnant, non–smoker, body mass index (BMI) <25, no risk factors for metabolic disease, non–vegetarian, normal bowel movements, no history of gastrointestinal disease, no use of antibiotics six months, pre– or probiotics three months prior to fecal donation, and no history of immunosuppressive or chemotherapeutic treatment. The donor collected feces using Protocult collection containers (Ability Building Center, Rochester, MN, USA). After collection, fecal samples were stored at room temperature along with a Merck anaerocult bag and treated at −80 °C within 3 h prior to storage. A fecal slurry of 10% (*w:v*) feces was prepared by homogenizing 40 g of fecal sample with 360 mL of sterile phosphate buffer solution (0.1 mol/L, pH 7.0) in an anaerobic glove box. The phosphate buffer solution consisted of NaH_2_PO_4_ [0.58% (*w:v*)] Na_2_HPO_4_ × 2H_2_O [1.03% (*w:v*)] and sodium thioglycolate solution [3.45% (*v:v*)]. After autoclaving (121 °C, 15 min), sterile glycerol 17% (*v:v*) was added. Homogenization and removal of solid particles were performed using a Stomacher^®^lab blender (Lab–blender 400, Seward Medical, London, UK) for 3 min. The sterile filter bags (Bagpage^®^R/25 400 mL, VWR International, Haasrode, Belgium) consisting of a whole side filter were able to filter the sludge and remove particulate food material. 500 mL of the fecal pool was filled into sterile 25–mL plastic vials and stored at −80 °C prior to use. 

### 4.5. Cultivation of the Fecal Slurry Suspension

Prior to use in the GIDM colon, the fecal slurry was cultured. The composition of the basal growth medium was phosphate buffer. The medium was autoclaved at 121 °C for 15 min. In the globe box (0.7% O_2_, 5% CO_2_, 5% H_2_, and 90% N_2_ at 35–37 °C), 20 mL of pooled frozen feces was thawed (37 °C, 8 min) and then 180 mL of sterile phosphate buffer was added. The bacterial suspension was incubated for 1 h before being added to the small intestinal digestion retentates with constant mixing using a magnetic stirrer.

### 4.6. GastroIntestinal Dialysis Model with Colon Phase, GIDM–Colon 

#### 4.6.1. Gastric Stage

In simulating the gastric stage for each individual experiment, 15 mg HTyr, 15 mg Tyr (two replicates, negative control), and 75 mg chlorogenic acid (positive control) were dissolved in 33.5 mL of high purity deionized water along with 16.5 mL pepsin solution (2052.6 U/mL digest) and adjusted to a pH of 2 with 6 M HCl. Samples were incubated in a shaking water bath at 37 °C (120 strokes/min) for 1 h. After the gastric phase, 1.5 mL of the sample was collected and stored at −80 °C for further analysis. No dialysis was performed during the gastric phase, as absorption of phenols occurred in the intestinal phase.

#### 4.6.2. Small Intestinal Stage

In order to have a continuous dialysis flow of the metabolites mimicking the human gastrointestinal digestion, the contents of the gastric phase were immediately transferred manually into ultrafiltration cells (Amicon stirred cells) with dialysis membrane to simulate the small intestine phase, and 50 mL of high purity deionized water was added to obtain a total volume of 100 mL. Dialysis membranes were soaked in 0.1 mol/L NaOH (30 min) and washed three times with high–purity deionized water (10 min each time) before use. Four dialysis cells were interconnected using pressure bottom switches. These on/off switches regulate the flow of oxygen–free N_2_ or water to each cell. The push–bottom control switch (the gas or liquid switching valve) is connected to a water tank that regulates pressure through a gas/liquid switch. This switch is responsible for supplying H_2_O (pressure is adjusted indirectly at the cells) or gas (pressure is adjusted directly at the cells) to the cells and mimics the transport from the lumen to mucosa (this occurs naturally when hyperosmotic solutions are delivered from the stomach to the duodenum). This model simulates only the passive diffusion of digested nutrients or bioactive compounds. A small dialysis bag containing an amount of 1 M NaHCO_3_ corresponding to the titratable acidity was added to each cell. The titratable acidity is the number of equivalents of NaOH (0.5 M used for its stability) required to titrate the amount of gastrointestinal digest to a pH of 7.0. The ultrafiltration cells were placed in a water bath (35–37 °C), continuously stirred and connected to a water tank and an N_2_ gas supply via pressure switches at the bottom. The N_2_ gas pressurizes the cells (2 bar) to allow dialysis. After 30 min of dialysis, 15 mL of a pancreatic bile solution was added to each cell. Dialysis was performed for a total of 2 h. After the small bowel phase, 1.5 mL samples of the retentate (the compounds not absorbed in the small bowel phase) and dialysate (the compounds absorbed in the small bowel phase) fractions were collected in pre–weighed vials in triplicate and stored at −80 °C for further analysis.

#### 4.6.3. Colonic Stage

To simulate the colonic stage, the pH of the retentate samples was adjusted to 5.8–6.0 with 1 M HCl, and the ultrafiltration cells were transferred to an anaerobic glove box. To each ultrafiltration cell, 50 mL of a 10% (*v:v*) fecal slurry suspension was added, except for the negative control sample. Instead, 50 mL of a sterile phosphate buffer solution was added to the negative control sample. The ultrafiltration cells were continuously shaken, and pressure was applied to the ultrafiltration cells (0.8 bar N_2_) to achieve dialysis. After 2, 4, 6, and 24 h, samples (1.5 mL) of the retentate and dialysate fractions were taken and stored at −80 °C.

### 4.7. HPLC Analysis of 6 h Retentate Samples of Chlorogenic Acid Colonic Metabolism

The 6 h retentate samples from the colon and the fecal suspension of stability control chlorogenic acid were centrifuged at 14,000 rpm for 10 min after the addition of 0.5 mL of analytical grade MeOH. The resulting supernatant was analyzed by HPLC analysis using Thermo Fischer Spectra system consisting of SCM 1000 vacuum membrane degasser, P1000XR gradient pump, Autosampler AS3000, UV 2000 detector with double wavelength. Samples were analyzed on an Xselect CSH C18 analytical column (3 × 250 mm, 5 μm) with an Xselect CSH C18 guard column (3 × 20 mm) from Waters. Mobile phase A consisted of a mixture of 5% MeOH and 0.05% H_3_PO_4_ (*v:v*) and mobile phase B consisted of 80% methanol MeOH and H_3_PO_4_ 0.05% (*v:v*). The gradient used was as follows: From 0 to 2 min 93% A and 7% B, from 2 to 50 min 20% A and 80% B, from 50 to 52min 20% A and 80% B, from 52 to 55 min 93% A and 7% B. The run time was 66 min, the injection volume was 20 μL and the flow rate was 0.3 mL/min. Quantification of chlorogenic acid and its metabolites was performed at different wavelengths according to the absorption maximum. Therefore, chlorogenic acid, homovanillic acid, and caffeic acid were analyzed at 310 nm and 3–phenylpropionic acid at 210 nm. The compounds were identified on the basis of retention time, UV spectrum and spiking with commercially available relevant standards. In both experiments with HTyr and Tyr, the chlorogenic acid was metabolized according to the key metabolites expected after 6 h, confirming the proper metabolic activity by the cultured bacteria, the precisely adjusted pH, enzyme, and temperature conditions.

### 4.8. Sample Pretreatment Prior Analysis, Quality Control Samples and Data Acquisition

All retentate samples were dissolved with 0.5 mL of analytical grade MeOH and centrifuged (4 °C, 14,000 rpm, 10 min). The supernatants were evaporated under vacuum and centrifugation at 25 °C until completely dry and immediately stored at −20 °C. All dialysate samples were immediately stored at −80 °C and freeze–dried to dryness in a freeze dryer (Labconco FreeZone 18 L, Kansas City, MO, USA) and immediately stored at −20 °C. Pooled samples were prepared as equal aliquots (10 uL) from the 80 samples tested, thoroughly mixed, and the mixture was then prepared in the same manner as the final samples (see Section 4.8). In addition, an external analytical quality control of rutin at 10 μg/mL was added at the beginning and end of the acquisition. QC samples were injected at the beginning of each analytical batch, one QC sample every ten samples during the run, and one QC sample at the end of the batch. To avoid instrumental problems, the ion transfer tube was removed and cleaned every 50 injections. All samples were in biological triplicate liquids, and the order of samples was randomized.

### 4.9. UPLC–-HRMS Analysis of Samples

Experiments to identify metabolites of HTyr biotransformation in the stomach, intestine, and colon were performed using Orbitrap. Eluants, column temperature, and column properties were investigated for the development of UPLC conditions. ACN resulted in better peak shape, along with acidified water and column temperature of 40 °C. An Acquity UPLC peptide BEH C18 (100 × 2.1 mm, 1.7 μm) was used. All samples were analyzed using an LC gradient consisting of H_2_O with 0.1% formic acid (FA) (solvent A) and ACN (solvent B). A general chromatographic and spectrometric method was developed based on pooled samples containing aliquots of all time points and compartments per compound. Elution was performed at a flow rate of 0.4 mL/min. The gradient program was set as follows: 2% B (0–1 min), 2–26% B (1–10 min), 26–65% B (10–16 min), 65–100% B (16–18 min), 100% B (18–20 min), 100–2% B (20–21 min), 2% B (21–25 min). The LC method was slightly changed for Tyr samples as follows 5% B (0–1 min), 2–26% B (1–10 min), 26–65% B (10–16 min), 65–100% B (16–18 min), 100% B (18–20 min), 100–5% B (20–21 min), 5% B (21–25 min). Measurements were performed with a total acquisition time of 25 min and a flow rate of 400 μL/min. The injection volume was 10 μL, the autosampler temperature was 7 °C, and the column temperature was at 40 °C. Mass spectra were recorded in negative ESI ion mode using an electrospray ionization (ESI) source. The capillary temperature was set at 350 °C, the capillary voltage at −30 V, and the tube lens at −100 V. Sheath and auxiliary gasses were set to 40 and 10 arb, respectively. Mass spectra were acquired in full scan mode in the range of 115–1000 *m/z*, with a resolving power of 30,000 at 500 *m/z* and a scan rate of 1 microscan per second. HRMS/MS experiments were performed using the data–dependent method with a collision energy of 35.0% (q = 0.25). The system was externally calibrated every fifty injections.

### 4.10. Statistical Process and Chemometrics

An automated data analysis workflow was used for unbiased screening of metabolites. For this purpose, commercially available software and free open–source software were combined with the developed workflow. All UPLC–MS injections of HTyr and Tyr were recorded using Xcalibur 2.2. Raw files (raw, Thermo). A non–targeted screening workflow was applied using the MZmine and R software packages. Raw data were uploaded to MZmine 2.53, which is integrated with ADAP (Automated Data Analysis Pipeline) peak peaking. While this is a step in the direction of reducing false positive and false negative chromatographic peaks, it gives confidence in the direction of fully automated data preprocessing [69]. All information is included in Table S1 (Supporting Information). These lists were exported as a .csv file and imported into SIMCA 14.1 software (Umetrics, Sweden) for statistical analysis. Mainly PCA was implemented for visualization of the samples. The generated models were evaluated according to their R2 and Q2 parameters, which indicate the measure of fit and predictability, respectively. Only models with R2 values close to 1, Q2 values above 0.5, or models with lower R2 but close to Q2 were accepted. All peaks evaluated as possible metabolites were registered in Table 1 and Table 2, along with identification information. In addition, the HRMS and HRMS/MS spectra of the identified metabolites are also listed in the supporting information.

### 4.11. Identification Workflow

Firstly, UPLC–HRMS chromatograms and their corresponding HRMS spectra (<2 ppm) were examined. The extraction–ion method was used in parallel with peak–to–peak selection to obtain the corresponding full–scan spectra. The proposed elemental composition (EC) of each detected peak along with isotopic patterns and ring double bond equivalents (RDBeq) were used to confirm the proposed structures. Moreover, HRMS/MS spectra contributed to the identification of specific chemical properties based on internal databases. In addition, online databases were used for additional structural information. Identification criteria were established as follows: (a) Maximum mass deviation of ±5 ppm between theoretical and measured parent ions, (b) product ions must not exceed the maximum mass deviation of ±10 ppm, (c) the identified biotransformation products were not present in the blank or negative control sample, (d) the biotransformation product was present in both replicates at a given sampling time.

## 5. Conclusions

In the present study, HTyr and Tyr metabolism was investigated using the *in vitro* continuous flow GIDM–colon model, which mimics human GI metabolism. This particular model provides a continuous dialysis flow that simulates passive diffusion from the lumen to the mucosa under controlled conditions, i.e., pH, oxygen level, basic enzymes, and gut bacteria that simulate metabolism in the stomach, small intestine, and colon. In this study, the GIDM–colon model was mainly used to investigate the interactions of the gut microbiota with the phenolic alcohols HTyr and Tyr during a constant 24 h colonic metabolism. However, significant conclusions have been drawn for stomach and small intestine conditions and their impact on the availability and metabolic fate of HTyr and Tyr, at least in the current *in vitro* model. The methodology used with UPLC–HRMS and HRMS/MS analysis, dereplication tools for the identification of metabolites in the three different compartments, as well as MVA with unsupervised methods, offered valuable insight towards a more holistic perspective. Obviously, the catechol group played a key role in the metabolic fate of parent compound HTyr and its metabolites. The *ortho*–hydroxyl group of HTyr seems to promote autooxidation reactions through the formation of *ortho*–quinones, which trigger a sequential chain of reactions leading to a variety of metabolites. Another important finding of the present study is the detection of dimers and trimers, especially for HTyr and to a much lesser extent for Tyr, which most likely are formed through autooxidation pathways. Finally, Tyr metabolites are degraded by the microflora of the colon in a similar manner as HTyr. For the first time, the polymerization of phenolic alcohols during their metabolism was established and proposed as autooxidation reaction thereof. To our knowledge, this is the first study reporting a complete list with full spectrometric data of the metabolic derivatives of HTyr and Tyr, along with the possible mechanisms of their chemical transformations in the different conditions of the GI tract.

## Figures and Tables

**Table 1:** Chromatographic and spectrometric information of HTyr identified metabolites.

Νο	RT (min)	EC	Exp. [M−H]^−^	Theor. [M−H]^−^ *m/z*	Δm (ppm)	RDB eq.	Fragments MS/MS	Compartments								Metabolites
			*m/z*					S0H	S1H	SID	SIR	C2H	C4H	C6H	C24H	
**HTyr1**	3.27	C_8_H_9_O_3_	153.05617	153.05462	−0.20	4.5	123	+	+	t	t	+	+	+	+	**HTyr**
**HTyr2**	4.39	C_8_H_7_O_2_	135.04541	135.0452	1.90	5.5	nd	–	+	–	–	–	–	–	+	**Quinone or epoxy or dihydro derivative of Tyr**
**HTyr3**	4.64	C_9_H_9_O_4_	181.05087	181.0506	1.32	5.5	nd	–	–	–	–	–	+	+	+	**Dihydrocaffeic acid**
**HTyr4**	4.70	C_7_H_5_O_3_	137.02500	137.02514	1.40	5.5	nd	–	+	+	–	t	t	–	–	**Hydroxybenzoic acid**
**HTyr5**	4.72	C_8_H_7_O_3_	151.0408	151.0401	4.6	5.5	123,103	+	+	t	–	+	+	+	+	**DOPAL (3** **,** **4 dihydroxyphenyl acetaldehyde)**
**HTyr** **6**	5.51	C_9_H_7_O_3_	163.0405	163.0401	2.50	6.5	nd	–	–	–	–	–	–	–	+	**Coumaric acid**
**HTyr** **7**	6.19	C_8_H_7_O_4_	167.0428	167.0428	0.23	5.5	nd	–	–	–	–	+	+	+	+	**DOPAC (dihydroxyphenylacetic acid)**
**HTyr8**	6.24	C_9_H_11_O_4_	183.0665	183.0663	1.01	4.5	nd	–	–	–	–	+	+	+	+	**4–(** **1** **,3–dihydroxypropyl) benzene–1,2–diol**
**HTyr9**	6.38	C_8_H_7_O_2_	135.0454	135.0452	2.1	5.5	nd	–	+	+	+	+	+	+	+	**PPA (phenylacetic acid)**
**HTyr10**	6.26	C_9_H_7_O_4_	179.0351	179.0350	0.54	6.5	nd	–	–	+	+	+	+	+	+	**Caffeic acid**
**HTyr** **1** **1**	6.48	C_16_H_17_O_9_	353.0872	353.0878	−1.65	8.5	235,265,247	–	–	+	–	–	–	–	–	**Hydroxylated dimer of HTyr (*homodimer*)**
**HTyr** **1** **2**	6.98	C_7_H_5_O_2_	121.0299	121.0295	2.96	5.5	nd	–	t	t	t	t	t	t	t	**Benzoic acid**
**HTyr** **1** **3**	8.03	C_16_H_17_O_4_	273.0766	273.0768	−0.79	9.5	nd	–	–	–	–	+	+	+	+	**Dimer of HTyr (*heterodimer*)**
**HTyr** **1** **4**	8.11	C_16_H_15_O_8_	335.0766	335.0772	−1.90	9.5	nd	–	–	+	+	+	–	–	–	**Hydroxylated dimer of HTyr (*homodimer*)**
**HTyr15**	8.35	C_16_H_17_O_6_	305.1028	305.1031	−0.91	8.5	275,179,161	–	+	–	–	–	–	–	–	**Dimer of HTyr (*homodimer*)**
**HTyr16**	8.75	C_9_H_9_O_3_	165.05594	165.05462	1.30	5.5	147	–	–	–	–	–	+	+	+	**Hydroxyphenyl propionic acid**
**HTyr17**	8.78	C_9_H_7_O_2_	147.0454	147.0454	1.64	6.5	119	–	–	–	–	–	+	+	+	**Dehydrated hydroxyphenyl propionic acid**
**HTyr18**	8.78	C_8_H_7_O	119.0505	119.0502	2.38	5.5	nd	–	–	–	–	–	–	+	+	**Phenylacetaldehyde**
**HTyr19**	9.20	C_10_H_11_O_2_	163.0767	163.0767	1.3	5.5	nd	–	t	–	–	–	–	–	–	**Phenylethyl acetate or 4–(4–hydroxyphenyl)–2–oxobutane**
**HTyr** **2** **0**	9.20/ 10.29	C_23_H_23_O_10_	459.1291	459.1297	−1.33	12.5	nd	–	–	+	+	–	–	–	–	**Trimer of HTyr (*homodimer*)**
**HTyr** **2** **1**	9.22–11.74	C_11_H_13_O_3_	193.0871	193.0870	0.49	5.5	163	–	+	–	–	–	–	–	–	**4–methoxyphenethyl acetate or 4–(3–hydroxy–4–methoxy–phenyl)–butan–2–one**
**HTyr** **2** **2**	9.24/ 9.53/ 10.35/10.75	C_23_H_23_O_9_	443.1341	443.1348	−1.49	12.5	247,399	–	–	+	+	–	–	–	–	**Hydroxylated trimer of HTyr (*heterodimer*)**
**HTyr** **2** **3**	3.52/9.87	C_10_H_11_O_4_	195.06632	195.0663	0.17	5.5	nd	–	+	–	–	+	+	+	+	**Hydroxytyrosol acetate**
**HTyr** **2** **4**	9.97	C_10_H_11_O_5_	211.0611	211.0612	−0.40	5.5	nd	–	–	–	–	+	+	–	–	**Hydroxylated** **Hydroxytyrosol acetate**
**HTyr25**	8.12/ 11.29	C_16_H_15_O_6_	303.0870	303.0874	−1.43	9.5	167,123	–	+	–	+	+	+	+	+	**Hydroxylated dimer of HTyr**
**HTyr26**	11.75	C_16_H_17_O_5_	289.10779	289.10779	0.73	8.5	135,153	+	+	t	–	+	+	+	+	**Dimer of HTyr (*heterodimer*)**
**HTyr27**	12.71	C_23_H_21_O_9_	441.1185	441.1191	−1.30	13.5	nd	–	–	+	–	–	–	–	–	**Trimer of HTyr–2H** **(*homodimer*)**

**RT**: Retention Time; **EC**: Elemental Composition, **RDBeq.**: Ring Double Bond equivalent; t: traces; nd: not detected.

**Table 2:** Chromatographic and spectrometric information of Tyr metabolites.

Νο	RT (min)	EC	Exp. [M−H]^−^	Theor. [M−H]^−^	Δm (ppm)	RDBeq.	Fragments MS/MS	Compartments								Metabolites
			*m/z*					S0H	S1H	SID	SIR	C2H	C4H	C6H	C24H	
**Tyr1**	3.80	C_8_H_7_O_2_	135.0455	135.04552	2.58	5.5	nd	−	−	−	−	−	−	−	+	**PPA (phenylacetic acid)**
**Tyr2**	3.86	C_9_H_9_O_4_	181.0509	181.0506	1.39	5.5	nd	−	−	−	−	−	−	*t	+	**Dihydroxyphenylpropionic acid**
**Tyr3**	3.91	C_9_H_7_O_3_	163.0404	163.0401	2.16	6.5	nd	−	−	−	−	−	−	−	+	**Coumaric acid**
**Tyr4**	4.40	C_8_H_5_O_3_	149.0248	149.0244	2.84	6.5	nd	+	+	+	−	+	+	+	−	**Phenylpropionic acid**
**Tyr5**	4.45	C_7_H_5_O_2_	121.0299	121.0295	2.96	5.5	nd	+	+	+	+	−	+	+	+	**Benzoic acid**
**Tyr6**	4.50	C_24_H_25_O_6_	409.1657	409.1657	0.04	12.5	273	−	−	+	−	−	−	−	−	**Tyrosol Trimer**
**Tyr7**	4.53	C_16_H_17_O_4_	273.1133	273.1132	0.29	8.5	243,213,137	+	+	+	+	–-	+	+	+	**Tyrosol (ESI source Dimer)**
**Tyr8**	5.21	C_16_H_17_O_5_	289.1082	289.1081	0.13	8.5	271,121	−	−	+	+	+	+	+	+	**Hydroxylated Tyrosol Dimer**
**Tyr9**	3.84	C_8_H_7_O	119.0505	119.0502	2.38	5.5	nd	−	−	−	−	−	−	+	+	**Phenylacetaldehyde**
**Tyr10**	6.89	C_9_H_9_O_3_	165.05594	165.05462	1.30	5.5	147	−	−	−	−	−	+	+	+	**Hydroxyphenylpropionic acid**
**Tyr11**	6.90	C_9_H_7_O_2_	147.0456	147.0452	2.9	6.5	103,119	−	−	−	−	−	+	+	+	**Cinnamic acid**
**Tyr12**	8.45	C_7_H_5_O_3_	137.02493	137.02493	3.5	5.5	nd	+	+	−	−	+	−	−	−	**4** **−** **hydroxybezoic acid**
**Tyr13**	12.27	C_10_H_11_O_3_	179.0717	179.0714	1.86	5.5	nd	t	+	t	−	t	−	−	−	**Tyrosol acetate**

**RT**: Retention Time; **EC**: Elemental Composition, **RDBeq.**: Ring Double Bond equivalent. Due to different LC method of Tyr samples (Section 4.9), a shift of RT in common metabolites with HTyr was observed. *t: traces; nd: not detected.

## Data Availability

The data presented in this study are available on request from the corresponding author. The data are not publicly available due to ongoing research on the scientific topic regarding metabolism of olive compounds.

## Supplementary Materials

The following supporting information can be downloaded at: https://www.mdpi.com/article/10.3390/metabo12050391/s1, Figure S1: The continuous dialysis *in vitro* GIDM–Colon model set up; Figure S2: UPLC–HRMS analysis set up for untargeted metabolomics approach; Figure S3: Indicatives UPLC–ESI(-)HRMS chromatograms of negative control sample of C2H; Figures S4–S29: HRMS spectra, full scan and MS/MS of selected HTyr and Tyr metabolites; Table S1: Sample coding and collection time points; Table S2: Processing parameters of MZmine 2.53.

## References

[1] Marković A.K., Torić J., Barbarić M., Brala C.J. (2019). Hydroxytyrosol, tyrosol and derivatives and their potential effects on human health. Molecules.

[2] Gavahian M., Mousavi Khaneghah A., Lorenzo J.M., Munekata P.E.S., Garcia-Mantrana I., Collado M.C., Meléndez-Martínez A.J., Barba F.J. (2019). Health benefits of olive oil and its components: Impacts on gut microbiota antioxidant activities, and prevention of noncommunicable diseases. Trends Food Sci. Technol..

[3] Santangelo C., Vari R., Scazzocchio B., De Sanctis P., Giovannini C., D’Archivio M., Masella R. (2017). Anti-inflammatory Activity of Extra Virgin Olive Oil Polyphenols: Which Role in the Prevention and Treatment of Immune-Mediated Inflammatory Diseases?. Endocr. Metab. Immune Disord. Drug Targets.

[4] González-Santiago M., Fonollá J., Lopez-Huertas E. (2010). Human absorption of a supplement containing purified hydroxytyrosol, a natural antioxidant from olive oil, and evidence for its transient association with low-density lipoproteins. Pharmacol. Res..

[5] Panel E., Nda A. (2011). Scientific Opinion on the substantiation of health claims related to polyphenols in olive and protection of LDL particles from oxidative damage (ID 1333, 1638, 1639, 1696, 2865), maintenance of normal blood HDL cholesterol concentrations (ID 1639), mainte. EFSA J..

[6] Rodríguez-Morató J., Boronat A., Kotronoulas A., Pujadas M., Pastor A., Olesti E., Pérez-Mañá C., Khymenets O., Fitó M., Farré M. (2016). Metabolic disposition and biological significance of simple phenols of dietary origin: Hydroxytyrosol and tyrosol. Drug Metab. Rev..

[7] De La Torre R., Covas M.I., Pujadas M.A., Fitó M., Farré M. (2006). Is dopamine behind the health benefits of red wine?. Eur. J. Nutr..

[8] D’Angelo C., Franceschelli S., Quiles J.L., Speranza L. (2020). Wide Biological Role of Hydroxytyrosol: Possible Therapeutic and Preventive Properties in Cardiovascular Diseases. Cells.

[9] Boronat A., Mateus J., Soldevila-Domenech N., Guerra M., Rodríguez-Morató J., Varon C., Muñoz D., Barbosa F., Morales J.C., Gaedigk A. (2019). Cardiovascular benefits of tyrosol and its endogenous conversion into hydroxytyrosol in humans. A randomized, controlled trial. Free Radic. Biol. Med..

[10] De La Torre R. (2008). Bioavailability of olive oil phenolic compounds in humans. Inflammopharmacology.

[11] Heleno S.A., Martins A., Queiroz M.J.R.P., Ferreira I.C.F.R. (2015). Bioactivity of phenolic acids: Metabolites versus parent compounds: A review. Food Chem..

[12] Hakala K. (2008). Liquid Chromatography-Mass Spectrometry in Studies of Drug Metabolism and Permeability. https://helda.helsinki.fi/handle/10138/19166.

[13] Rein M.J., Renouf M., Cruz-Hernandez C., Actis-Goretta L., Thakkar S.K., da Silva Pinto M. (2013). Bioavailability of bioactive food compounds: A challenging journey to bioefficacy. Br. J. Clin. Pharmacol..

[14] Serreli G., Deiana M. (2018). Biological Relevance of Extra Virgin Olive Oil Polyphenols Metabolites. Antioxidants.

[15] Serreli G., Le Sayec M., Diotallevi C., Teissier A., Deiana M., Corona G. (2021). Conjugated metabolites of hydroxytyrosol and tyrosol contribute to the maintenance of nitric oxide balance in human aortic endothelial cells at physiologically relevant concentrations. Molecules.

[16] Atul Bhattaram V., Graefe U., Kohlert C., Veit M., Derendorf H. (2002). Pharmacokinetics and Bioavailability of Herbal Medicinal Products. Phytomedicine.

[17] Borah P., Hazarika S., Deka S., Venugopala K.N., Nair A.B., Attimarad M., Sreeharsha N., Mailavaram R.P. (2020). Application of Advanced Technologies in Natural Product Research: A Review with Special Emphasis on ADMET Profiling. Curr. Drug Metab..

[18] Koppel N., Rekdal V.M., Balskus E.P. (2018). Chemical transformation of xenobiotics by the human gut microbiota. Science.

[19] Possemiers S., Bolca S., Verstraete W., Heyerick A. (2011). The intestinal microbiome: A separate organ inside the body with the metabolic potential to influence the bioactivity of botanicals. Fitoterapia.

[20] Corona G., Spencer J., Dessì M. (2009). Extra virgin olive oil phenolics: Absorption, metabolism, and biological activities in the GI tract. Toxicol. Ind. Health.

[21] Vissers M.N., Zock P.L., Katan M.B. (2004). Bioavailability and antioxidant effects of olive oil phenols in humans: A review. Eur. J. Clin. Nutr..

[22] Suárez M., Romero M.P., Macià A., Valls R.M., Fernández S., Solà R., Motilva M.J. (2009). Improved method for identifying and quantifying olive oil phenolic compounds and their metabolites in human plasma by microelution solid-phase extraction plate and liquid chromatography-tandem mass spectrometry. J. Chromatogr. B Anal. Technol. Biomed. Life Sci..

[23] Kotronoulas A., Pizarro N., Serra A., Robledo P., Joglar J., Rubió L., Hernaéz Á., Tormos C., Motilva M.J., Fitó M. (2013). Dose-dependent metabolic disposition of hydroxytyrosol and formation of mercapturates in rats. Pharmacol. Res..

[24] Rodríguez-Morató J., Robledo P., Tanner J.A., Boronat A., Pérez-Mañá C., Oliver Chen C.Y., Tyndale R.F., de la Torre R. (2017). CYP2D6 and CYP2A6 biotransform dietary tyrosol into hydroxytyrosol. Food Chem..

[25] López de las Hazas M.C., Godinho-Pereira J., Macià A., Almeida A.F., Ventura M.R., Motilva M.J., Santos C.N. (2018). Brain uptake of hydroxytyrosol and its main circulating metabolites: Protective potential in neuronal cells. J. Funct. Foods.

[26] Mateos R., Goya L., Bravo L. (2005). Metabolism of the olive oil phenols hydroxytyrosol, tyrosol, and hydroxytyrosyl acetate by human hepatoma HepG2 cells. J. Agric. Food Chem..

[27] Lee D.H., Kim Y.J., Kim M.J., Ahn J., Ha T.Y., Lee S.H., Jang Y.J., Jung C.H. (2016). Pharmacokinetics of tyrosol metabolites in rats. Molecules.

[28] Miloš J., Belaj A., Pascual M., Sanz C., Miloš J. (2017). Handbook of Olive Oil: Phenolic Compounds, Production and Health Benefits.

[29] Mosele J.I., Martín-Peláez S., Macià A., Farràs M., Valls R.M., Catalán Ú., Motilva M.J. (2014). Faecal microbial metabolism of olive oil phenolic compounds: *In vitro* and in vivo approaches. Mol. Nutr. Food Res..

[30] Mosele J.I., Macià A., Motilva M.J. (2015). Metabolic and microbial modulation of the large intestine ecosystem by non-absorbed diet phenolic compounds: A review. Molecules.

[31] López De Las Hazas M.C., Piñol C., Macià A., Motilva M.J. (2017). Hydroxytyrosol and the Colonic Metabolites Derived from Virgin Olive Oil Intake Induce Cell Cycle Arrest and Apoptosis in Colon Cancer Cells. J. Agric. Food Chem..

[32] Ruiz-Garcia A., Bermejo M., Moss A., Casabo V.G. (2008). Pharmacokinetics in drug discovery. J. Pharm. Sci..

[33] Cheng F., Li W., Liu G., Tang Y. (2013). In Silico ADMET Prediction: Recent Advances, Current Challenges and Future Trends. Curr. Top. Med. Chem..

[34] Zhang T., Chen Q., Li L., Angela Liu L., Wei D.-Q. (2011). In Silico Prediction of Cytochrome P450-Mediated Drug Metabolism. Comb. Chem. High Throughput Screen..

[35] Manna C., Galletti P., Maisto G., Cucciolla V., D’Angelo S., Zappia V. (2000). Transport mechanism and metabolism of olive oil hydroxytyrosol in Caco-2 cells. FEBS Lett..

[36] D’Angelo S., Manna C., Migliardi V., Mazzoni O., Morrica P., Capasso G., Pontoni G., Galletti P., Zappia V. (2001). Pharmacokinetics and metabolism of hydroxytyrosol, a natural antioxidant from olive oil. Drug Metab. Dispos..

[37] Miro-Casas E., Covas M.I., Farre M., Fito M., Ortuño J., Weinbrenner T., Roset P., De La Torre R. (2003). Hydroxytyrosol disposition in humans. Clin. Chem..

[38] Robles-Almazan M., Pulido-Moran M., Moreno-Fernandez J., Ramirez-Tortosa C., Rodriguez-Garcia C., Quiles J.L., Ramirez-Tortosa M. (2018). Hydroxytyrosol: Bioavailability, toxicity, and clinical applications. Food Res. Int..

[39] Derendorf H. (2012). Pharmacokinetics of Natural Compounds. Planta Med..

[40] Breynaert A., Bosscher D., Kahnt A., Claeys M., Cos P., Pieters L., Hermans N. (2015). Development and Validation of an *in vitro* Experimental GastroIntestinal Dialysis Model with Colon Phase to Study the Availability and Colonic Metabolisation of Polyphenolic Compounds. Planta Med..

[41] Fedi A., Vitale C., Ponschin G., Ayehunie S., Fato M., Scaglione S. (2021). *In vitro* models replicating the human intestinal epithelium for absorption and metabolism studies: A systematic review. J. Control Release.

[42] Cassidy A., Minihane A.M. (2017). The role of metabolism (and the microbiome) in defining the clinical efficacy of dietary flavonoids. Am. J. Clin. Nutr..

[43] Landberg R., Manach C., Kerckhof F.-M., Minihane A.-M., Saleh R.N.M., De Roos B., Tomas-Barberan F., Morand C., Van de Wiele T. (2019). Future prospects for dissecting inter-individual variability in the absorption, distribution and elimination of plant bioactives of relevance for cardiometabolic endpoints. Eur. J. Nutr..

[44] Van Dooren I., Foubert K., Bijttebier S., Breynaert A., Theunis M., Exarchou V., Claeys M., Hermans N., Apers S., Pieters L. (2018). *In vitro* gastrointestinal biotransformation and characterization of a Desmodium adscendens decoction: The first step in unravelling its behaviour in the human body. J. Pharm. Pharmacol..

[45] Tuenter E., Bijttebier S., Foubert K., Breynaert A., Apers S., Hermans N., Pieters L. (2017). *In Vitro* and in Vivo Study of the Gastrointestinal Absorption and Metabolisation of Hymenocardine, a Cyclopeptide Alkaloid. Planta Med..

[46] Rivera-Mondragón A., Peeters L., Van A.A., Breynaert A., Caballero-George C., Pieters L., Hermans N., Foubert K. (2020). Simulated gastrointestinal biotransformation of chlorogenic acid, flavonoids, flavonolignans and triterpenoid saponins in cecropia obtusifolia leaf extract. Planta Med..

[47] Mortelé O., Iturrospe E., Breynaert A., Lammens C., Britto X.B., Malhotra-Kumar S., Jorens P., Pieters L., van Nuijs A.L.N., Hermans N. (2019). Chlorogenic Acid as a Model Compound for Optimization of an *In Vitro* Gut Microbiome-Metabolism Model. Proceedings.

[48] Aschenbrenner E.M., Weiss C.K., Landfester K. (2009). Enzymatic esterification in aqueous miniemulsions. Chem. A Eur. J..

[49] McDonald S., Prenzler P.D., Antolovich M., Robards K. (2001). Phenolic content and antioxidant activity of olive extracts. Food Chem..

[50] Alemán-Jiménez C., Domínguez-Perles R., Medina S., Prgomet I., López-González I., Simonelli-Muñoz A., Campillo-Cano M., Auñón D., Ferreres F., Gil-Izquierdo Á. (2020). Pharmacokinetics and bioavailability of hydroxytyrosol are dependent on the food matrix in humans. Eur. J. Nutr..

[51] Pereira-Caro G., Sarriá B., Madrona A., Espartero J.L., Escuderos M.E., Bravo L., Mateos R. (2012). Digestive stability of hydroxytyrosol, hydroxytyrosyl acetate and alkyl hydroxytyrosyl ethers. Int. J. Food Sci. Nutr..

[52] Napolitano A., De Lucia M., Panzella L., d’Ischia M. (2010). The Chemistry of Tyrosol and Hydroxytyrosol: Implications for Oxidative Stress. Olives and Olive Oil in Health and Disease Prevention.

[53] Camilleri M., Gores G.J. (2015). Therapeutic targeting of bile acids. Am. J. Physiol. Gastrointest. Liver Physiol..

[54] Heřmánková E., Žák A., Poláková L., Hobzová R., Hromádka R., Širc J. (2018). Polymeric bile acid sequestrants: Review of design, *in vitro* binding activities, and hypocholesterolemic effects. Eur. J. Med. Chem..

[55] Caruso D., Visioli F., Patelli R., Galli C., Galli G. (2001). Urinary excretion of olive oil phenols and their metabolites in humans. Metab.-Clin. Exp..

[56] Tuck K.L., Hayball P.J., Stupans I. (2002). Structural characterization of the metabolites of hydroxytyrosol, the principal phenolic component in olive oil, in rats. J. Agric. Food Chem..

[57] Khymenets O., Farré M., Pujadas M., Ortiz E., Joglar J., Covas M.I., De La Torre R. (2011). Direct analysis of glucuronidated metabolites of main olive oil phenols in human urine after dietary consumption of virgin olive oil. Food Chem..

[58] De Lucia M., Panzella L., Pezzella A., Napolitano A., D’Ischia M. (2006). Oxidative chemistry of the natural antioxidant hydroxytyrosol: Hydrogen peroxide-dependent hydroxylation and hydroxyquinone/o-quinone coupling pathways. Tetrahedron.

[59] Rubió L., Macià A., Valls R.M., Pedret A., Romero M.-P., Solà R., Motilva M.-J. (2012). A new hydroxytyrosol metabolite identified in human plasma: Hydroxytyrosol acetate sulphate. Food Chem..

[60] Vogna D., Pezzella A., Panzella L., Napolitano A., D’Ischia M. (2003). Oxidative chemistry of hydroxytyrosol: Isolation and characterisation of novel methanooxocinobenzodioxinone derivatives. Tetrahedron Lett..

[61] Fulcrand H., Cheminat A., Brouillard R., Cheynier V. (1994). Characterization of compounds obtained by chemical oxidation of caffeic acid in acidic conditions. Phytochemistry.

[62] Arakawa R., Yamaguchi M., Hotta H., Osakai T., Kimoto T. (2004). Product analysis of caffeic acid oxidation by on-line electrochemistry/ electrospray ionization mass spectrometry. J. Am. Soc. Mass Spectrom..

[63] Hotta H., Sakamoto H., Nagano S., Osakai T., Tsujino Y. (2001). Unusually large numbers of electrons for the oxidation of polyphenolic antioxidants. Biochim. Biophys. Acta Gen. Subj..

[64] Achat S., Rakotomanomana N., Madani K., Dangles O. (2016). Antioxidant activity of olive phenols and other dietary phenols in model gastric conditions: Scavenging of the free radical DPPH and inhibition of the haem-induced peroxidation of linoleic acid. Food Chem..

[65] Brooks S.J., Doyle E.M., O’Connor K.E. (2006). Tyrosol to hydroxytyrosol biotransformation by immobilised cell extracts of Pseudomonas putida F6. Enzyme Microb. Technol..

[66] Annunziata F., Contente M.L., Pinna C., Tamborini L., Pinto A. (2021). Biocatalyzed Flow Oxidation of Tyrosol to Hydroxytyrosol and Efficient Production of Their Acetate Esters. Antioxidants.

[67] Xie P., Fan L., Huang L., Zhang C. (2021). Oxidative polymerization process of hydroxytyrosol catalysed by polyphenol oxidases or peroxidase: Characterization, kinetics and thermodynamics. Food Chem..

[68] Roche M., Dufour C., Mora N., Dangles O. (2005). Antioxidant activity of olive phenols: Mechanistic investigation and characterization of oxidation products by mass spectrometry. Org. Biomol. Chem..

[69] Myers O.D., Sumner S.J., Li S., Barnes S., Du X. (2017). One Step Forward for Reducing False Positive and False Negative Compound Identifications from Mass Spectrometry Metabolomics Data: New Algorithms for Constructing Extracted Ion Chromatograms and Detecting Chromatographic Peaks. Anal. Chem..

