# Supplementary material for: Availability and Metabolic Fate of Olive Phenolic Alcohols Hydroxytyrosol and Tyrosol in the Human GI Tract Simulated by the In Vitro GIDM–Colon Model
Source: Metabolites. 2022 Apr 26;12(5):391. doi: 10.3390/metabo12050391 (PMC9144922; doi:10.3390/metabo12050391)
Supplement: Supplementary file 1 [file metabolites-12-00391-s001.zip › metabolites-1684493-supplementary.pdf]

Supplementary File of Article

Availability and Metabolic Fate of Olive Phenolic Alcohols Hydroxytyrosol and Tyrosol in the Human GI Tract Simulated by the In Vitro GIDM–Colon Model

Maria Eleni Sakavitsi [1], Annelies Breynaert [2], Theodora Nikou [1], Stef Lauwers [2], Luc Pieters [2], Nina Hermans [2,*] and Maria Halabalaki [1,*]

[1] Division of Pharmacognosy and Natural Products Chemistry, Department of Pharmacy, National and Kapodistrian University of Athens, 15771 Athens, Greece; msakavitsi@pharm.uoa.gr (M.E.S.); th-nikou@pharm.uoa.gr (T.N.)

[2] Natural Products & Food Research and Analysis (NatuRA), Department of Pharmaceutical Sciences, University of Antwerp, 2000 Antwerp, Belgium; annelies.breynaert@uantwerpen.be (A.B.); stef.lauwers@uantwerpen.be (S.L.); luc.pieters@uantwerpen.be (L.P.)

* Correspondence: nina.hermans@uantwerpen.be (N.H.); mariahal@pharm.uoa.gr (M.H.); Tel.: +32-32652732 (N.H.); +30-2107274781 (M.H.)

Table S1: Sample coding and collection time points

Codes of Samples	Compartment/Type of Samples	Time point
**S0H**	Stomach	T=0h
**S1H**	Stomach	T=1h
**SID**	Small Intestine Dialysates	T=1.5h
**SIR**	Small Intestine Retentates	T=1.5h
**C2H**	Colon Dialysates	T=2h
**C4H**	Colon Dialysates	T=4h
**C6H**	Colon Dialysates	T=6h
**C24H**	Colon Dialysates	T=24h

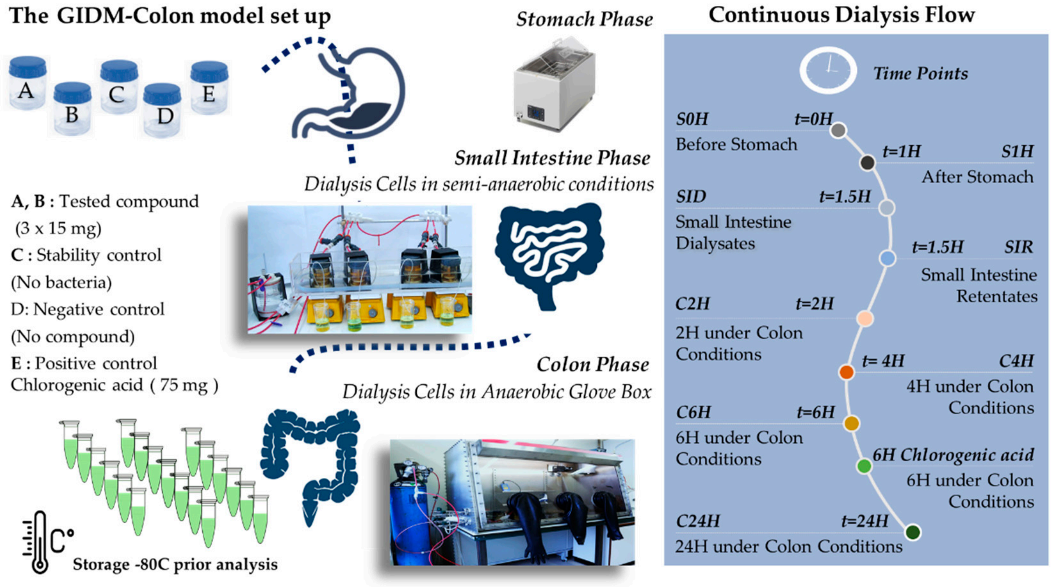

Figure S1: The continous dialysis *in vitro* GIDM-Colon model set up. **A,B cells**: 15mg of HTyr or Tyr; **C cells**: Stability control (15mg of HTyr or Tyr, no bacteria); **D cells**: Negative control (no compounds, with bacteria); **E cells**: Positive control (75mg of chlorogenic acid, no tested compounds, with bacteria); Time points: **S0H** are the samples collected at the time of administration of tested compounds and control samples at the gastric conditions. **S1H** are the samples collected after 1h exposure in gastric conditions. **SID** are the dialysate samples collected after 1.5h of their exposure to small intestine conditions that have followed passive diffusion by passing through the cell membrane. **SIR** are the retentate samples collected after 1.5h of their exposure to small intestine conditions that have not passed the cell membrane. The retentates of small intestine were subjected to colon phase by adding fecal bacteria and transfering them in anaerobic glove box. The collection for colon phase started 2h after the samples were exposed to colon conditions and passed the cell membranes.**C2H, C4H, C6H, C24H** are the dialysate samples after 2h, 4h, 6h, 24h exposed to colon conditions. Stomach and small intestine samples were collected in biological duplicates and colon samples in triplicates. All samples were stored to -80°C prior analysis.

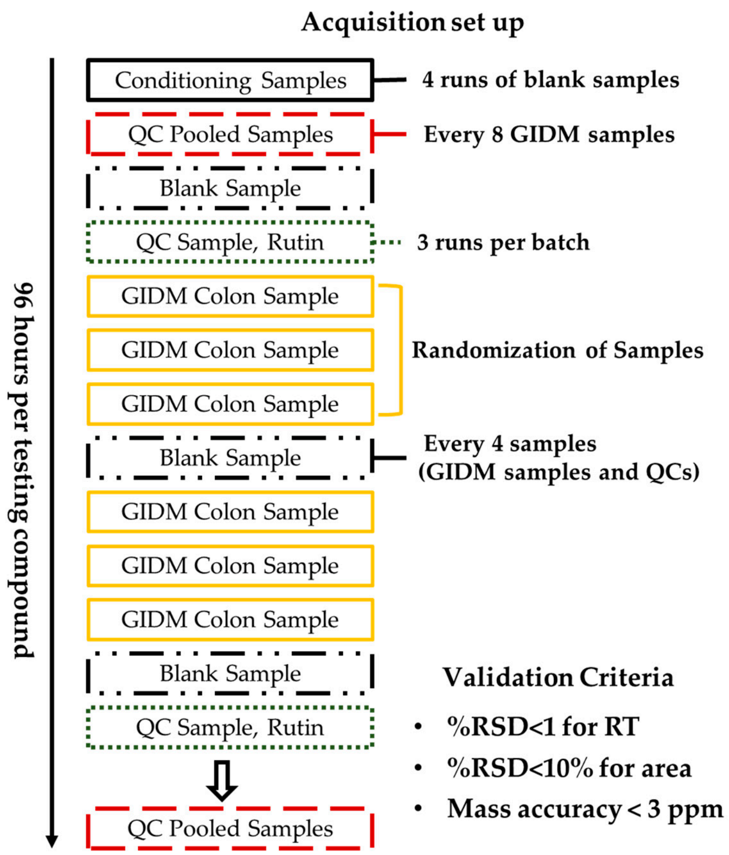

Figure S2*:* UPLC-HRMS analysis set up for untargeted metabolomics approach. *Conditioning, blank, quality control (QC)-pooled* (mixture of all GIDM-colon samples) and *analytical QC* (rutin 100ug/ml) samples were used. Randomisation of GIDM-colon samples was applied to avoid bias. Monitoring of selected peaks in QC-pooled samples were also considered throughout the aquisition to ensure system stability and repeatability as well as to avoid induced (technical) variability.

Table S2: Processing parameters of MZmine 2.53.

**MZmine 2.53**	Tyr	HTyr
**1.Mass Detection**		
RT	3-18min	3-18min
Noise Level	5E4	5E4
**2.Chromatogram Builder**		
Time span	0.03	0.03
**3.Chromatogram deconvolution**		
Algorithm	Local min search	Local min search
Peak duration	0.03-0.7min	0.03-1min
**4.Isotopic peaks grouper**		
m/z tolerance	0.001-5ppm	0.001-5ppm
RT tolerance	0.05min	0.05min
**5.Alignment**		
Algorithm	Join aligner	Join aligner
**6.Filtering: Feature lists row filter**		
Min peaks per row	2	2
**7.Gap Filling**		
Intensity tolerance	20%	20%

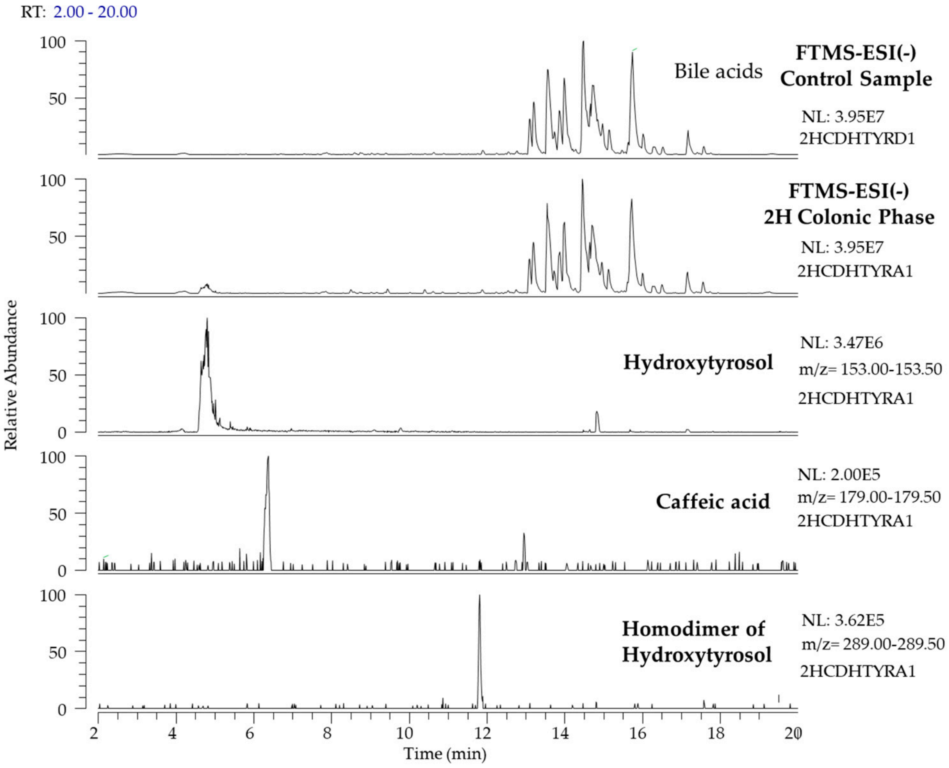

Figure S3: Indicatives UPLC-ESI(-)HRMS chromatograms of negative control sample of **C2H**; Base peak chromatogram of **C2H**; XIC chromatograms of **HTyr** (@*m/z* 153), **Caffeic acid** (@*m/z* 179) and Homodimer of HTyr (**HTyr26** @m/z 289).

HRMS spectra of Hydroxytyrosol (HTyr) and Tyrosol (Tyr) Metabolites

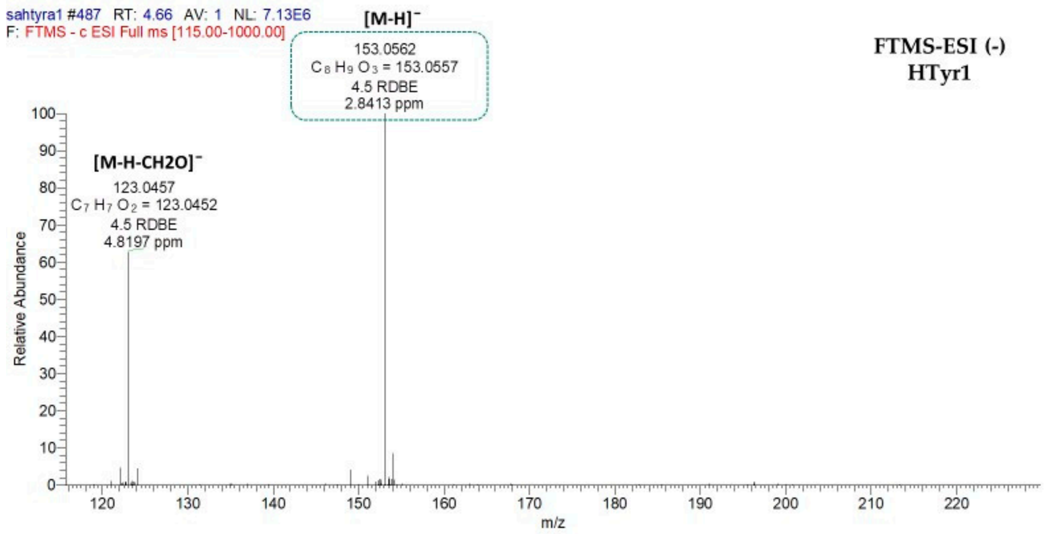

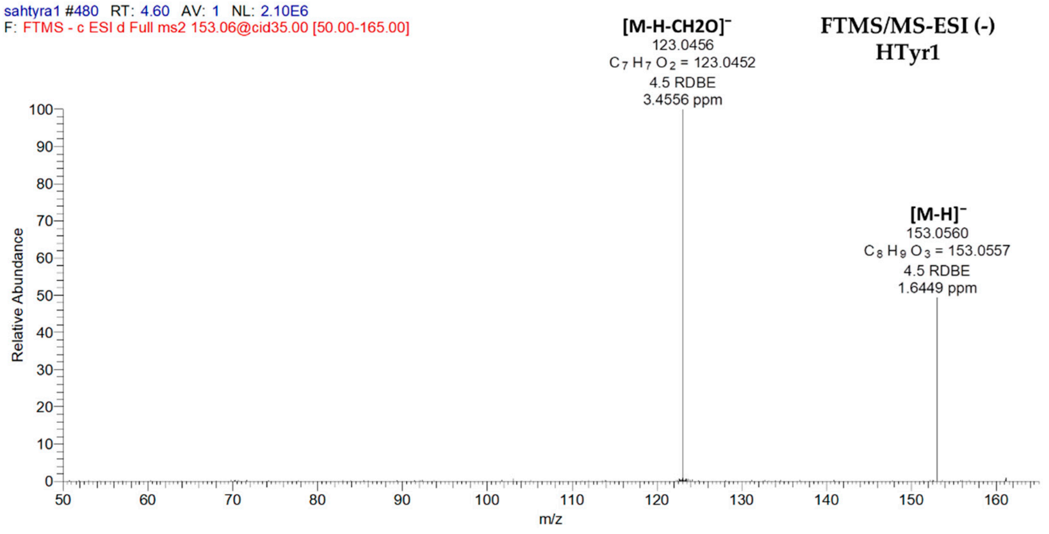

Figure S4: HRMS spectra, full scan (upper) and HRMS/MS (down) of **HTyr1.**

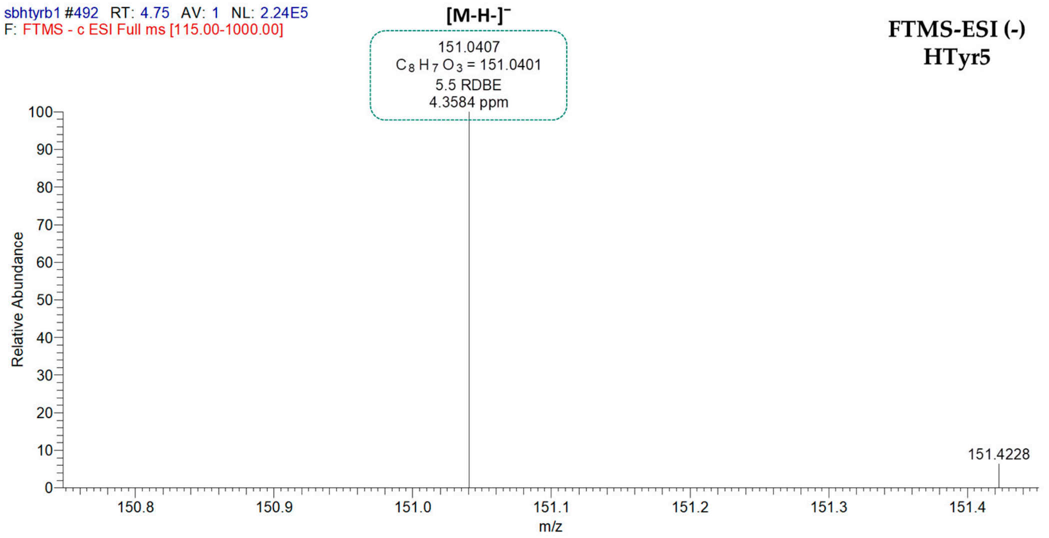

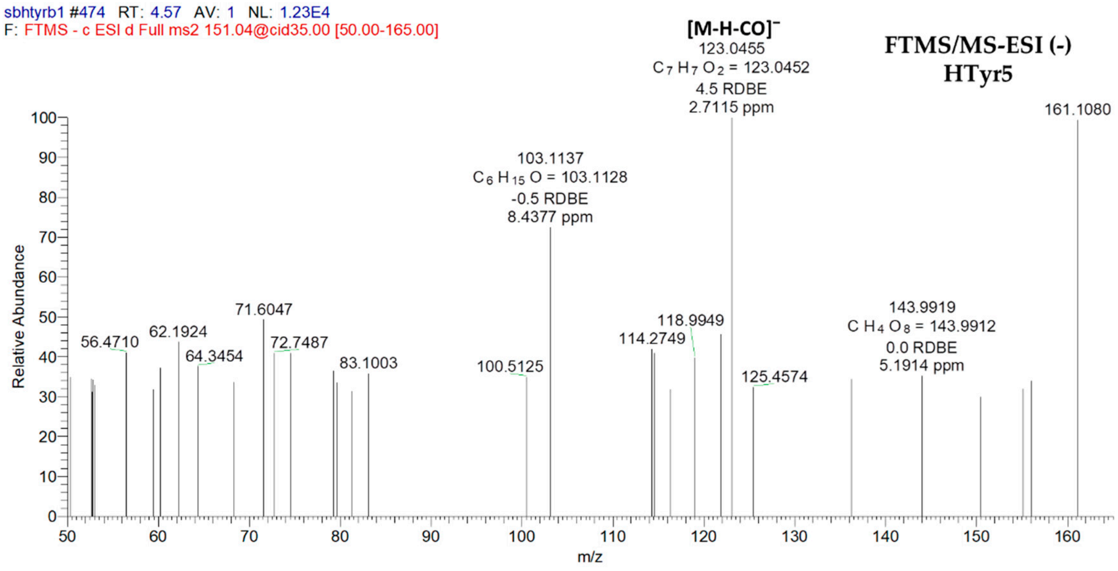

Figure S5: HRMS spectra, full scan (upper) and MS/MS (down) of **HTyr5.**

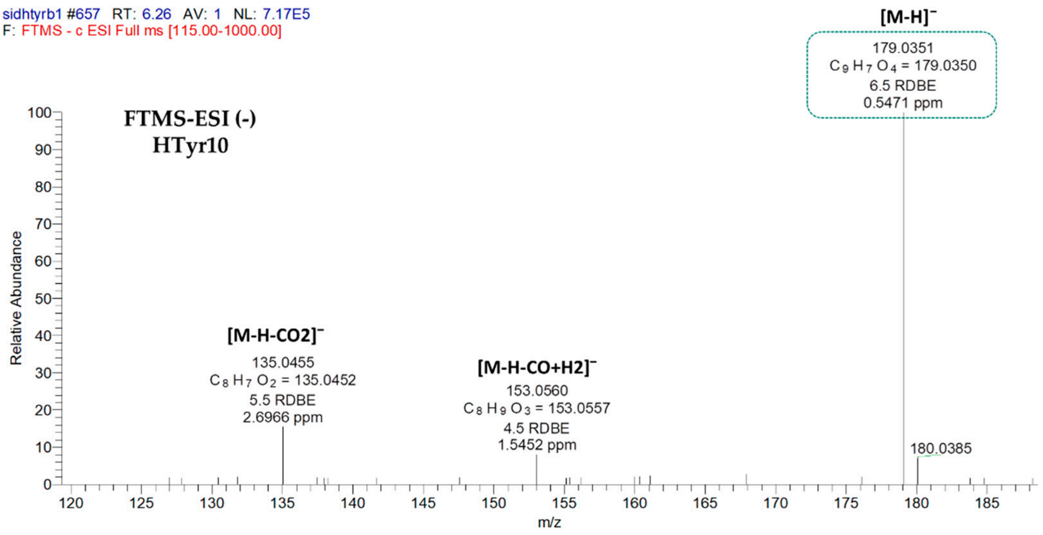

Figure S6: HRMS full scan spectrum of **HTyr10**.

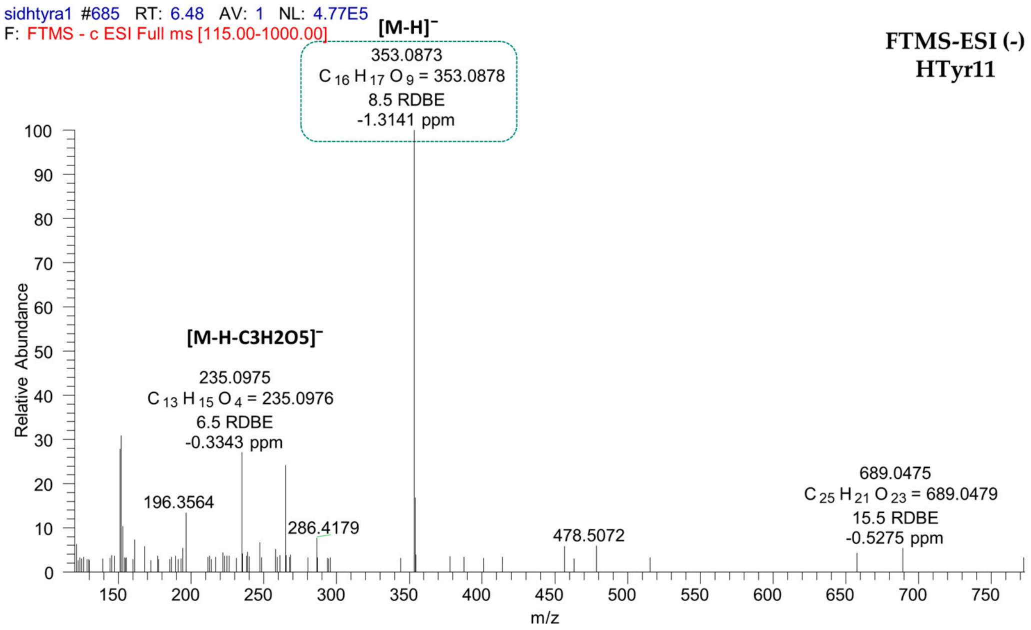

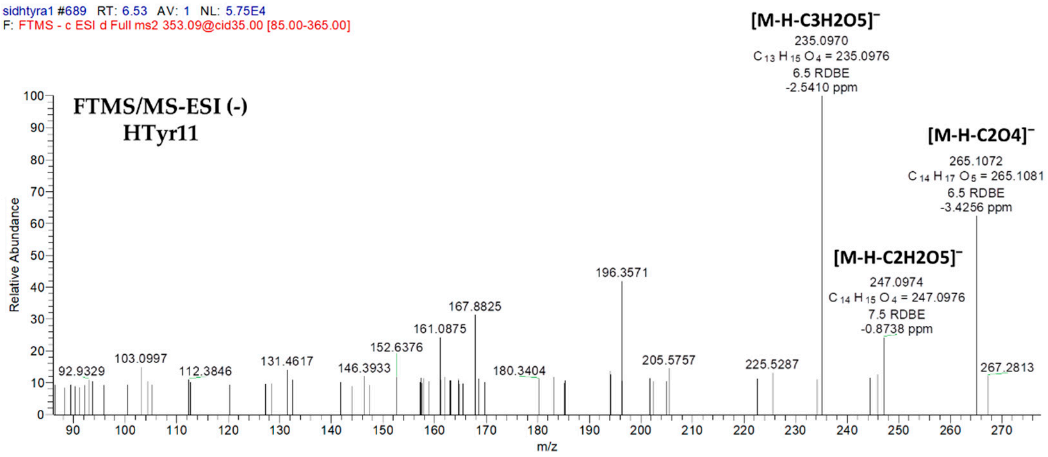

Figure S7: HRMS spectra, full scan (upper) and MS/MS (down) of **HTyr11.**

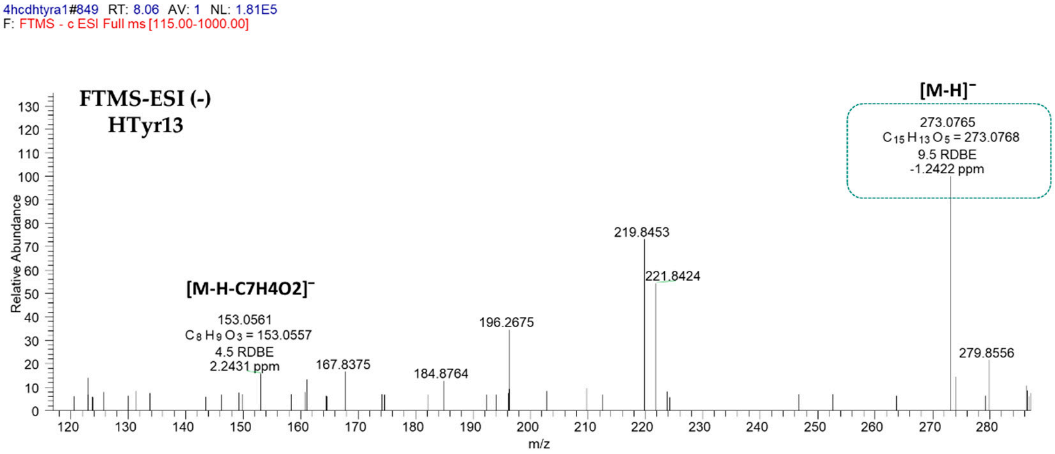

Figure S8: HRMS full scan spectrum of **HTyr13.**

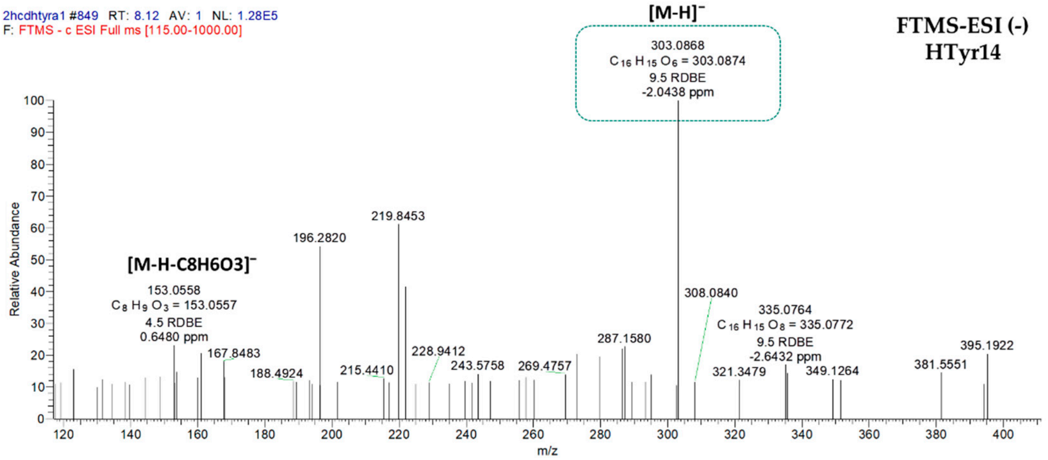

Figure S9: HRMS full scan spectrum of **HTyr14.**

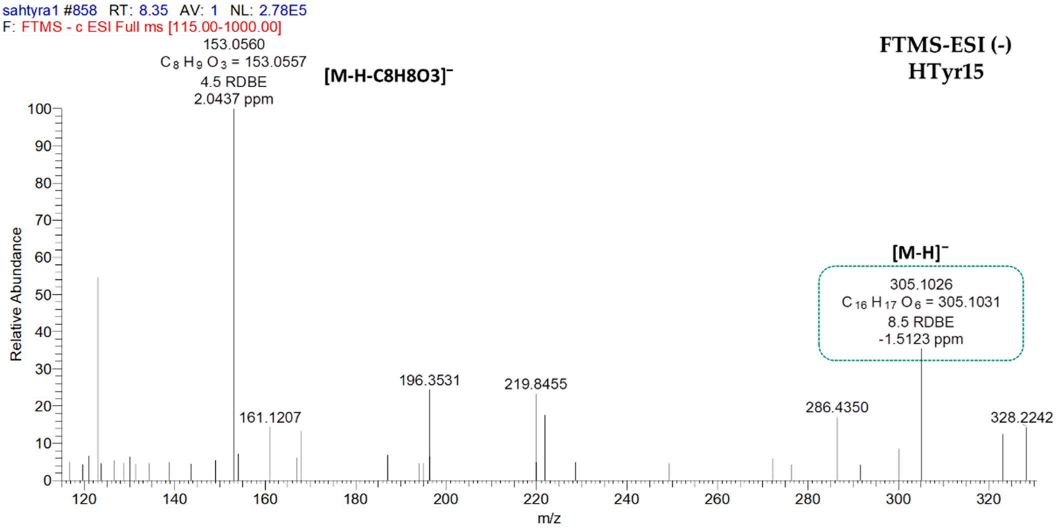

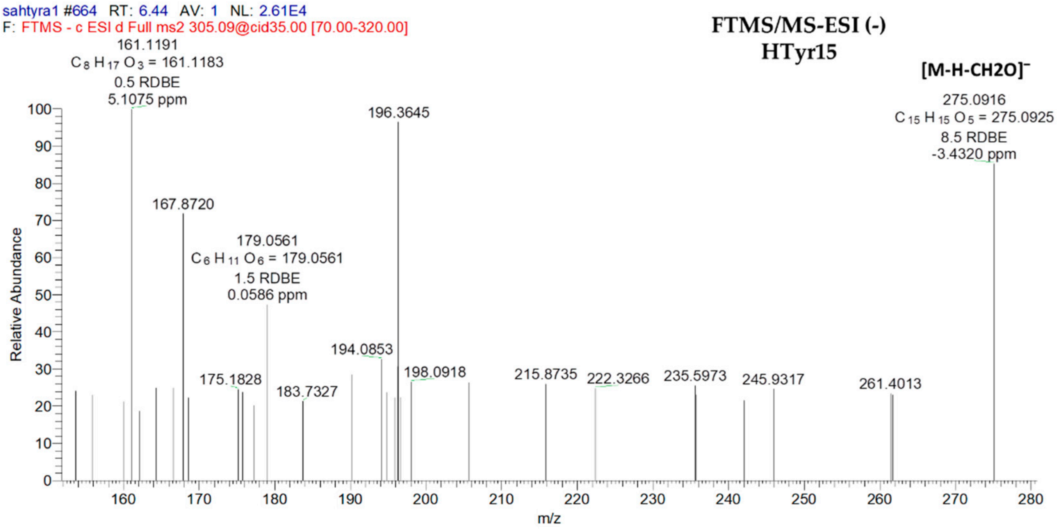

Figure S10: HRMS spectra, full scan (upper) and MS/MS (down) of **HTyr15.**

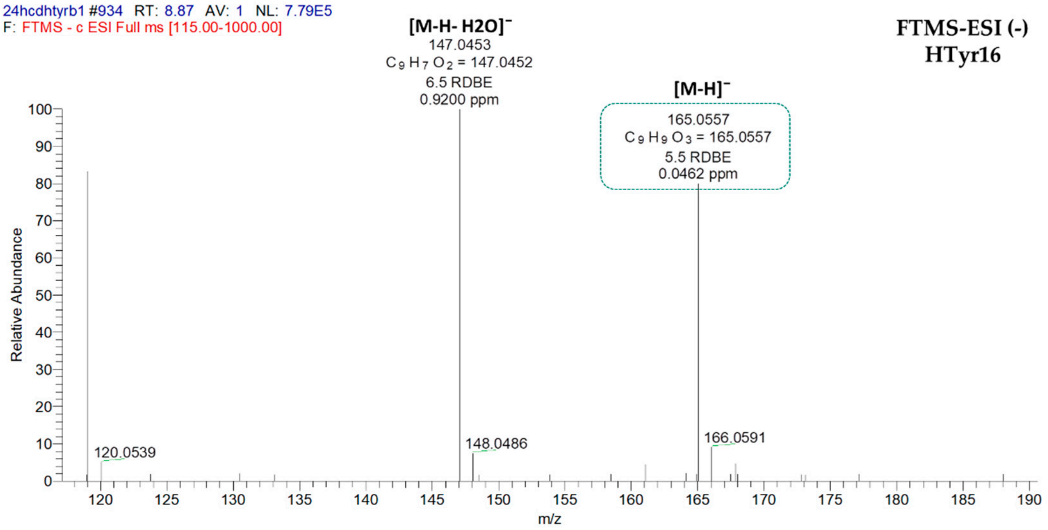

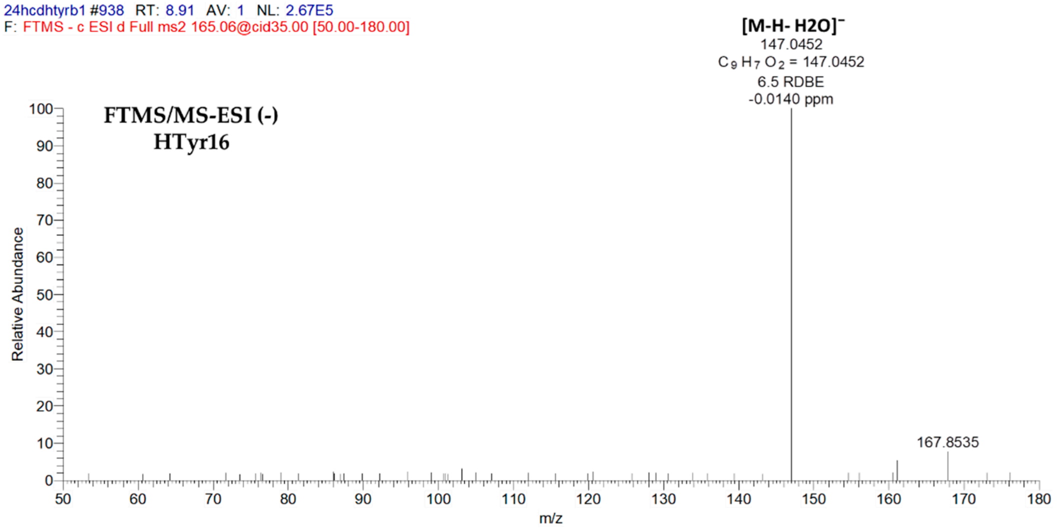

Figure S11: HRMS spectra, full scan (upper) and MS/MS (down) of **HTyr16**.

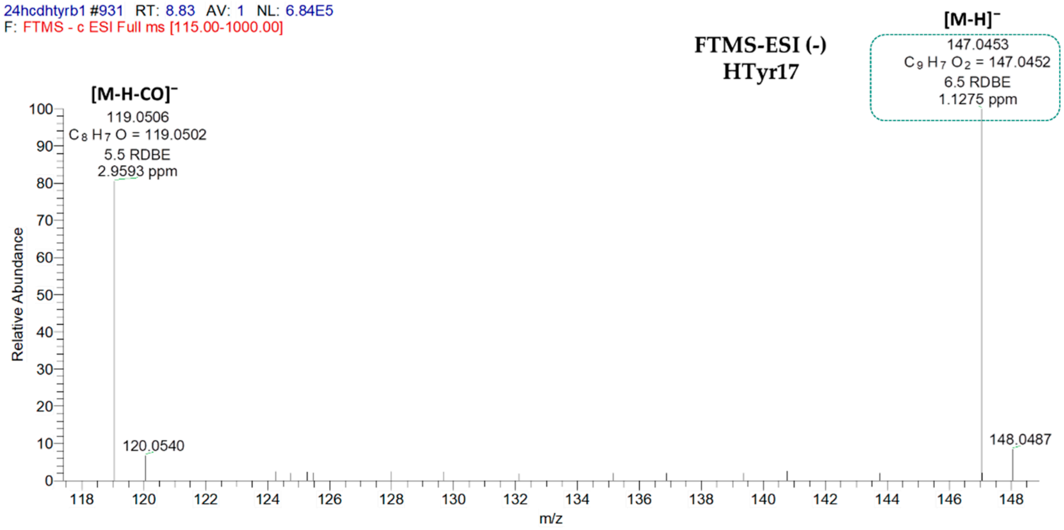

Figure S12: HRMS full scan spectrum of **HTyr17.**

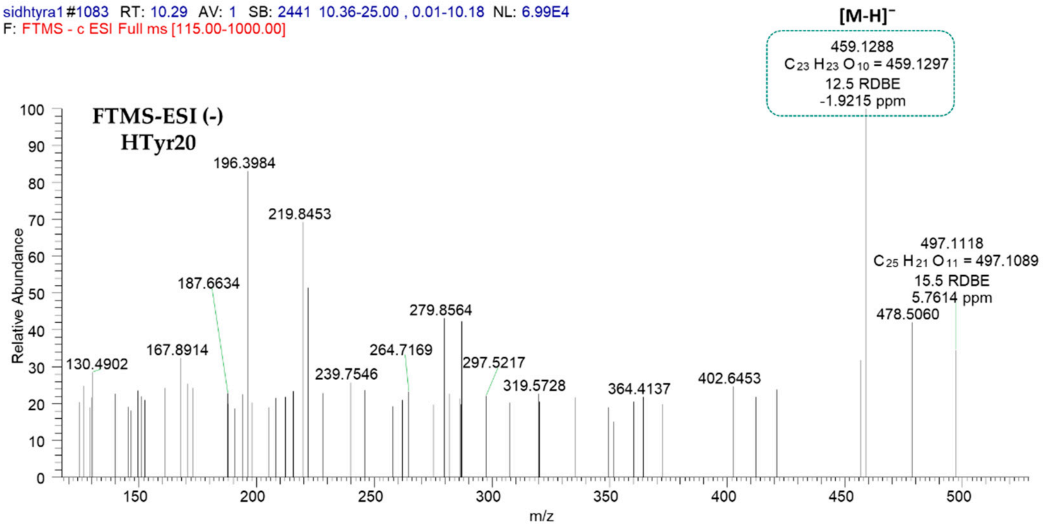

Figure S13: HRMS full scan spectrum of **HTyr20.**

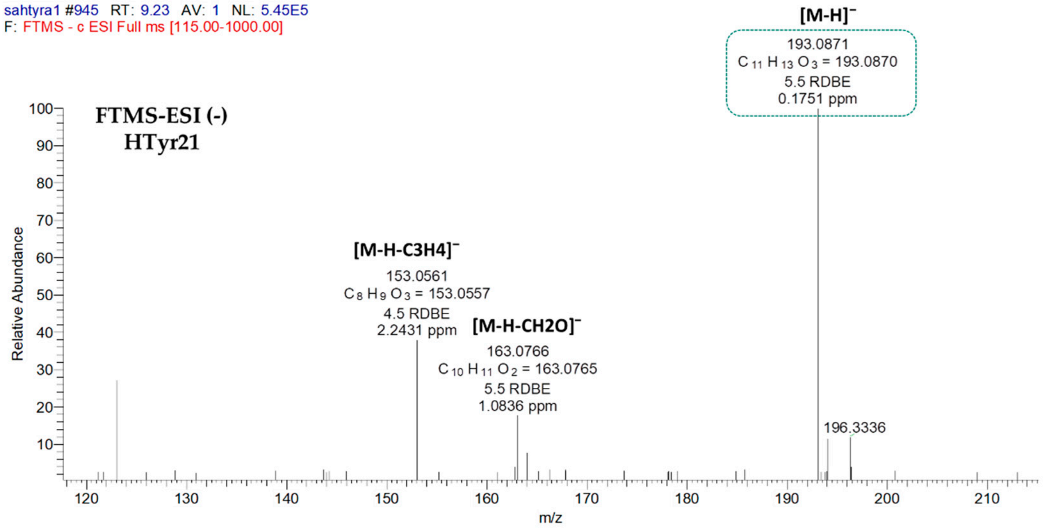

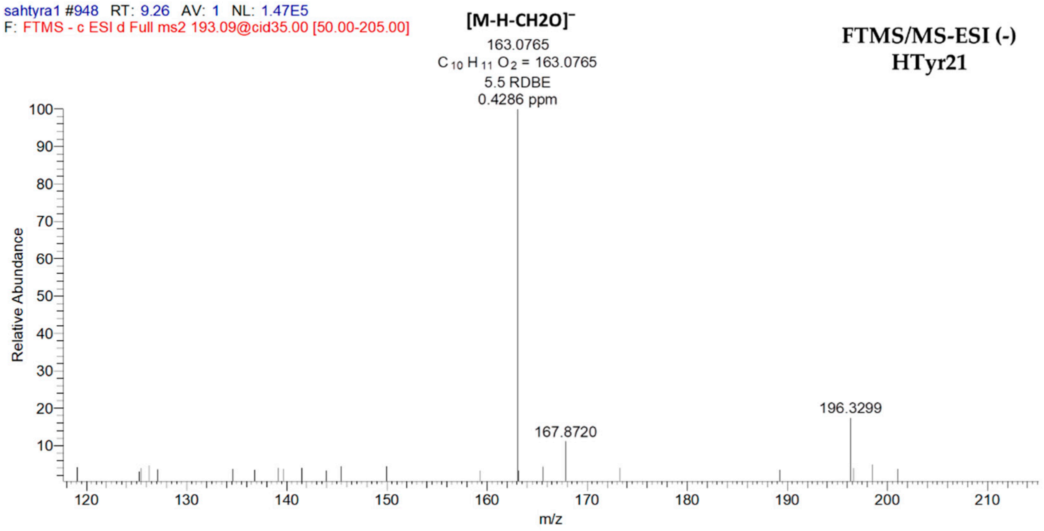

Figure S14: HRMS spectra, full scan (upper) and MS/MS (down) of **HTyr21**.

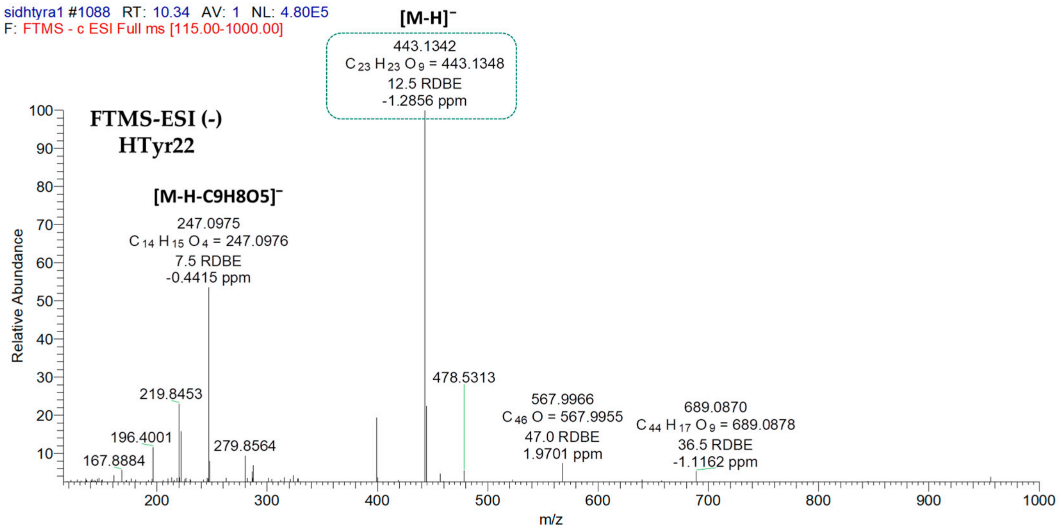

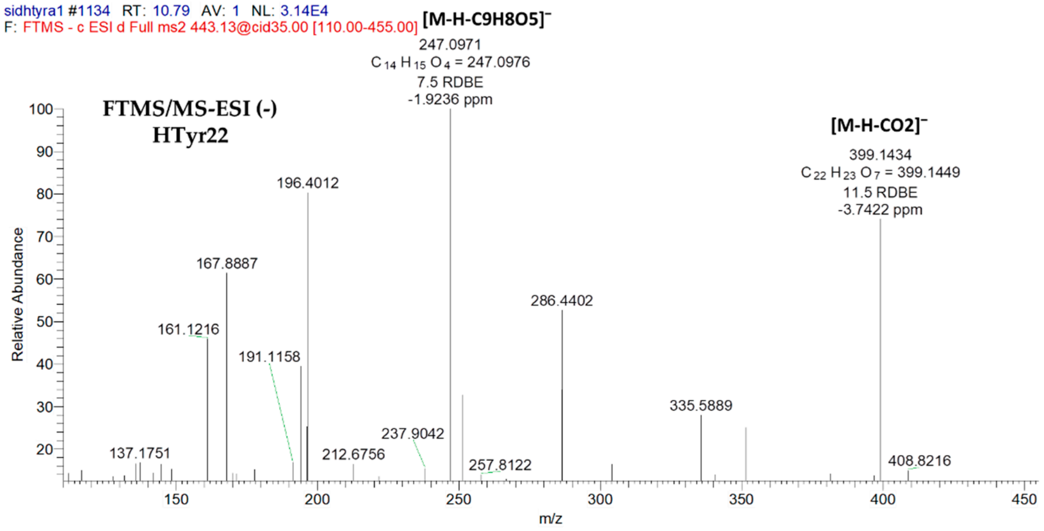

Figure S15: HRMS spectra, full scan (upper) and MS/MS (down) of **HTyr22**.

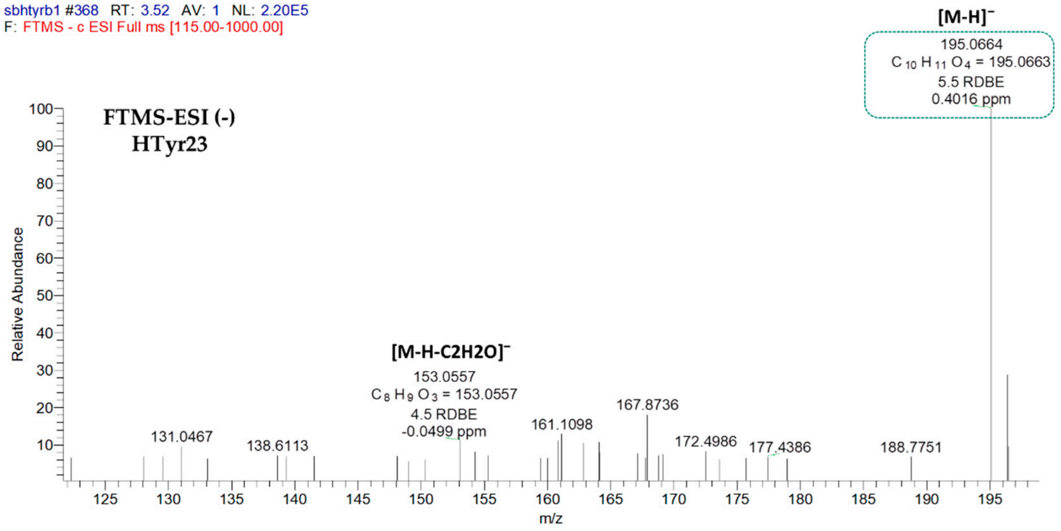

Figure S16: HRMS full scan spectrum of **HTyr23**.

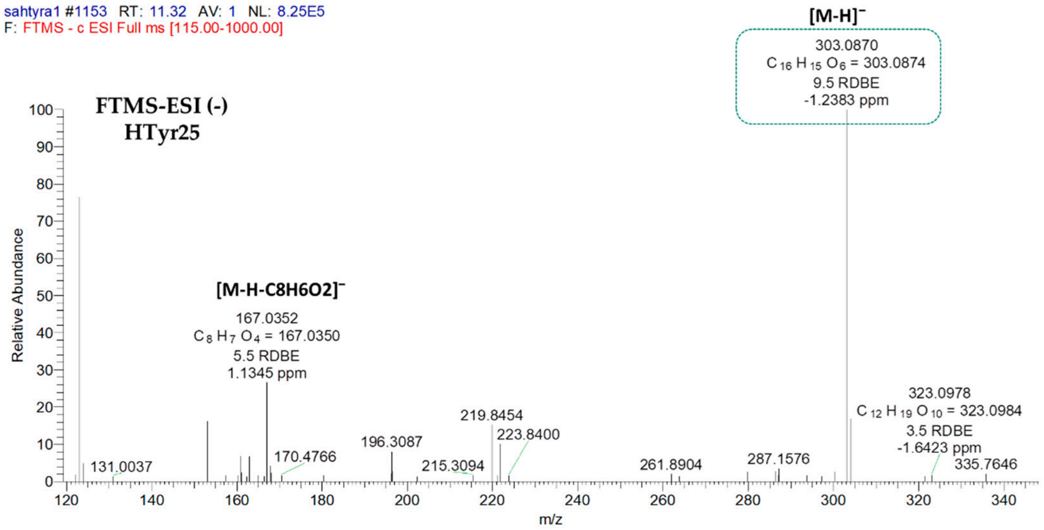

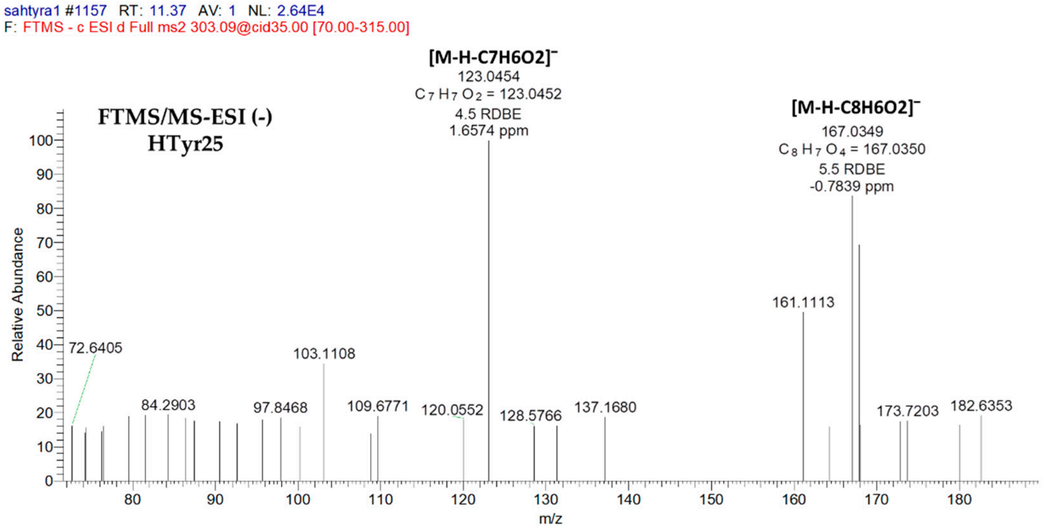

Figure S17: HRMS spectra, full scan (upper) and MS/MS (down) of **HTyr25**.

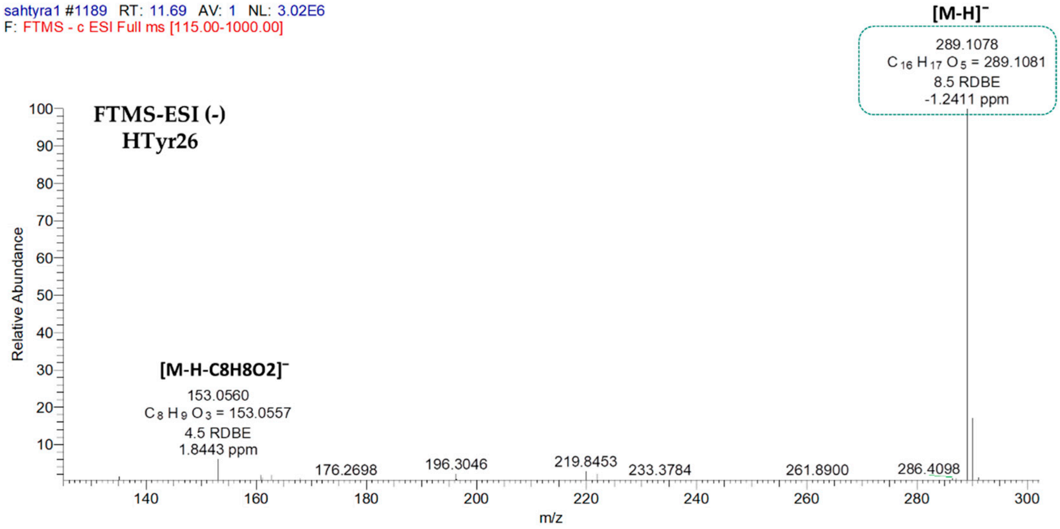

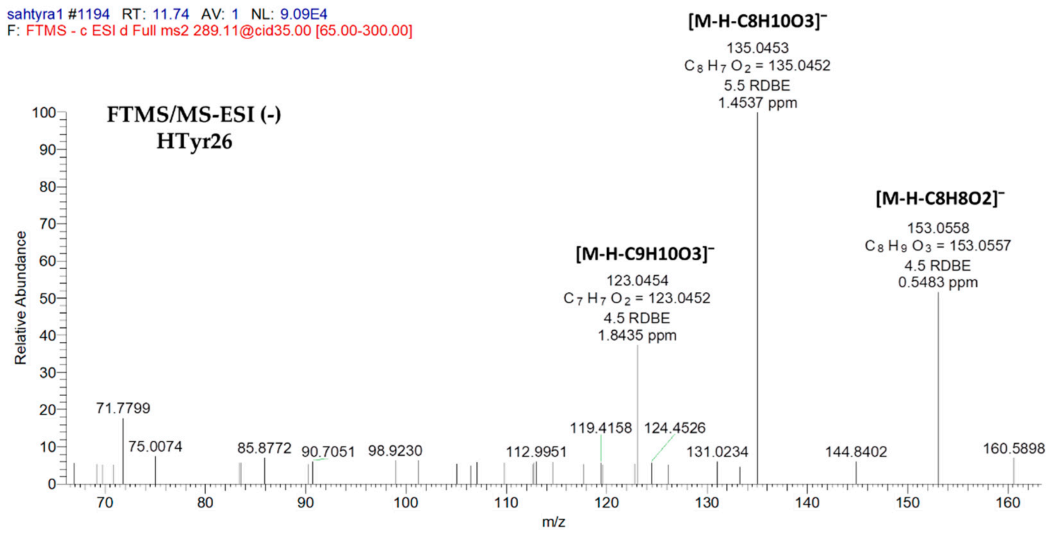

Figure S18: HRMS spectra, full scan (upper) and MS/MS (down) of **HTyr26.**

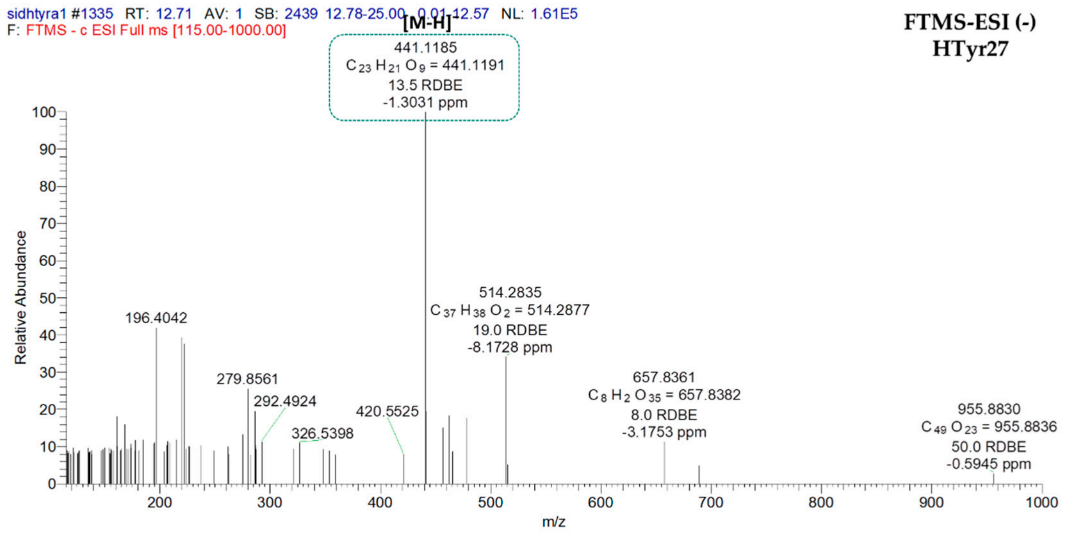

Figure S19: HRMS full scan spectrum of **HTyr27.**

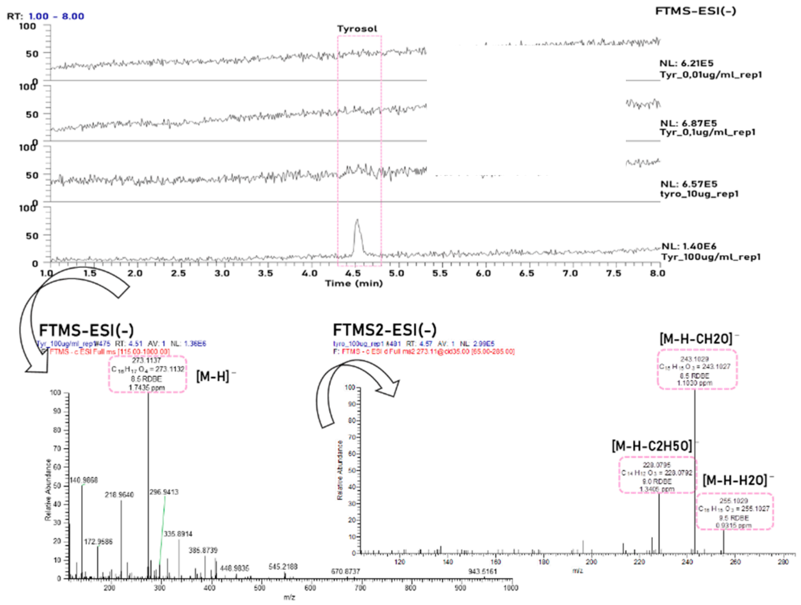

Figure S20: HRMS spectra, full scan of **Tyr1** in four concentrations (0.01ug/ml, 0.1ug/ml, 10ug/ml, 100ug/ml).

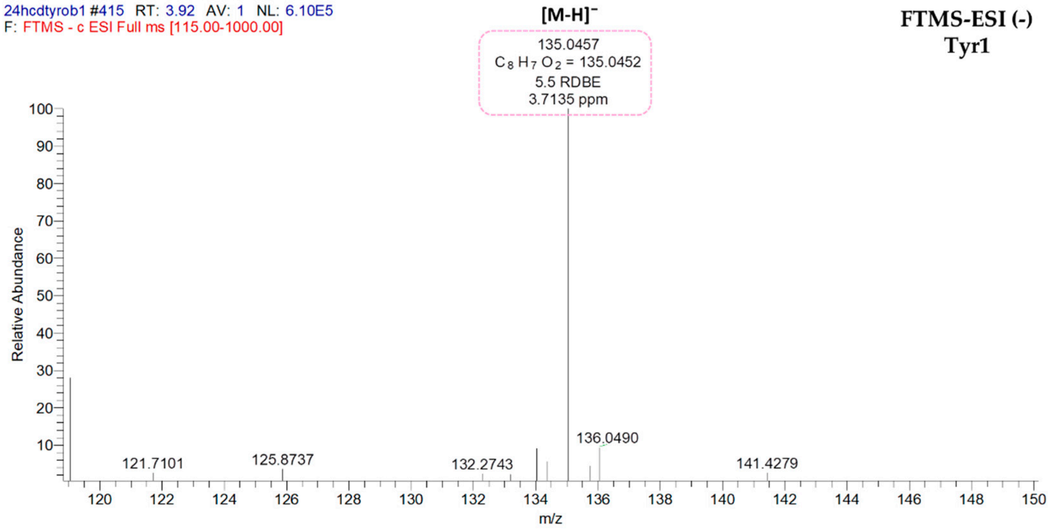

Figure S21: HRMS full scan spectrum of **Tyr1**.

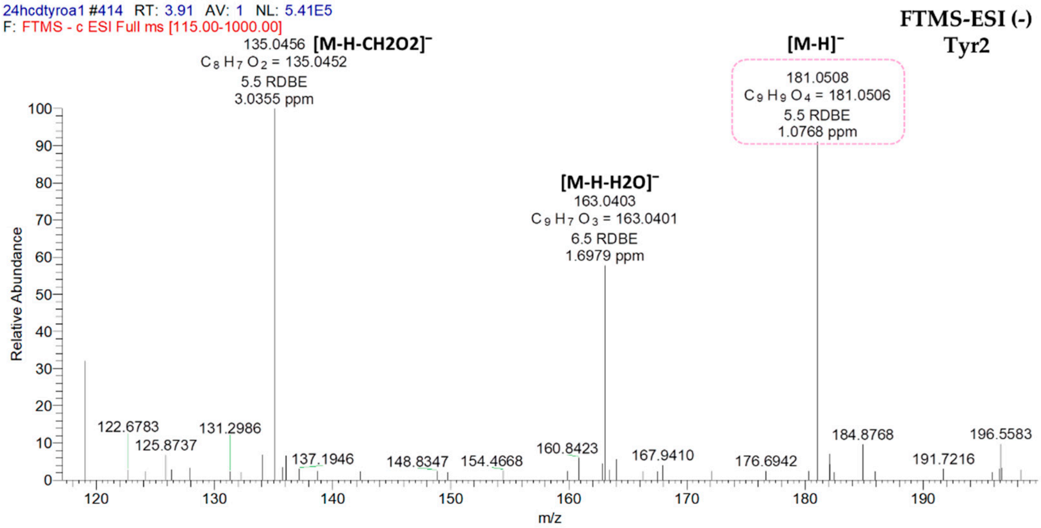

Figure S22: HRMS full scan spectrum of **Tyr2**.

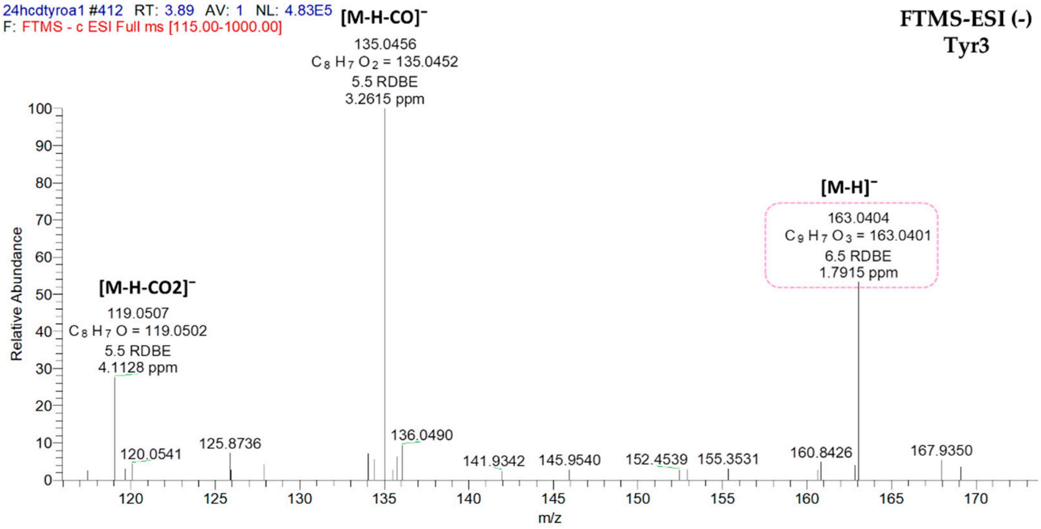

Figure S23: HRMS full scan spectrum of **Tyr3**.

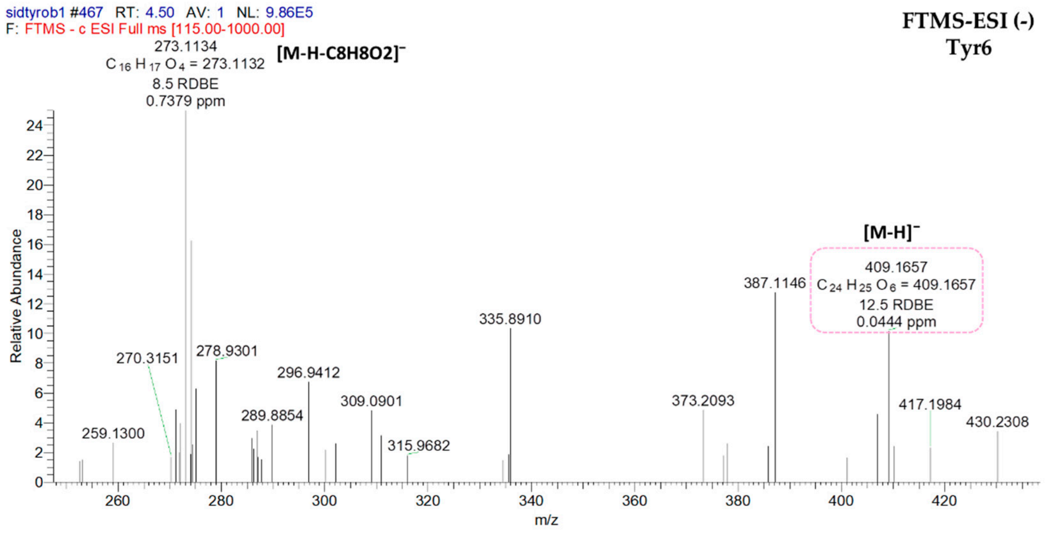

Figure S24: HRMS full scan spectrum of **Tyr6**.

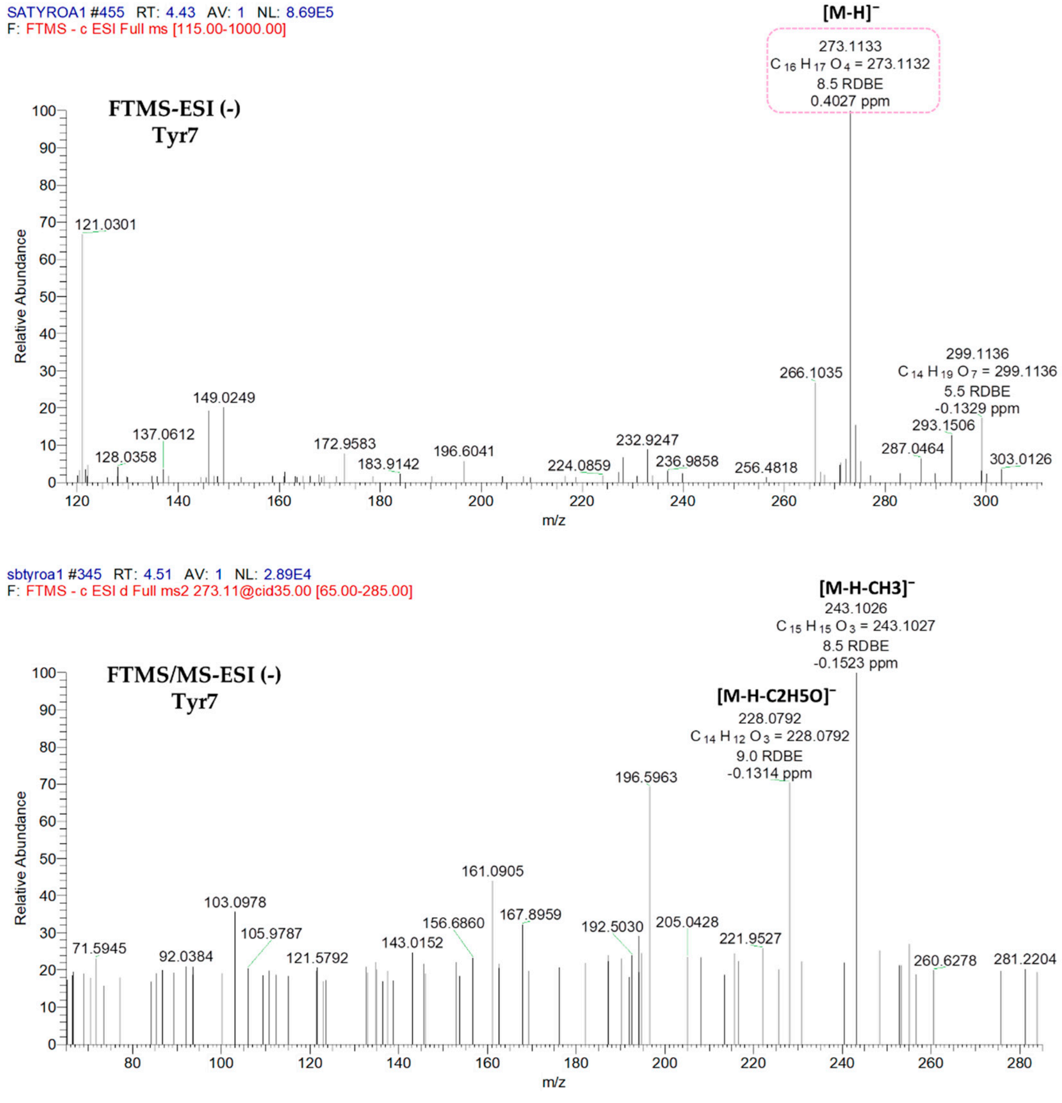

Figure S25: HRMS spectra, full scan (upper) and MS/MS (down) of **Tyr7**.

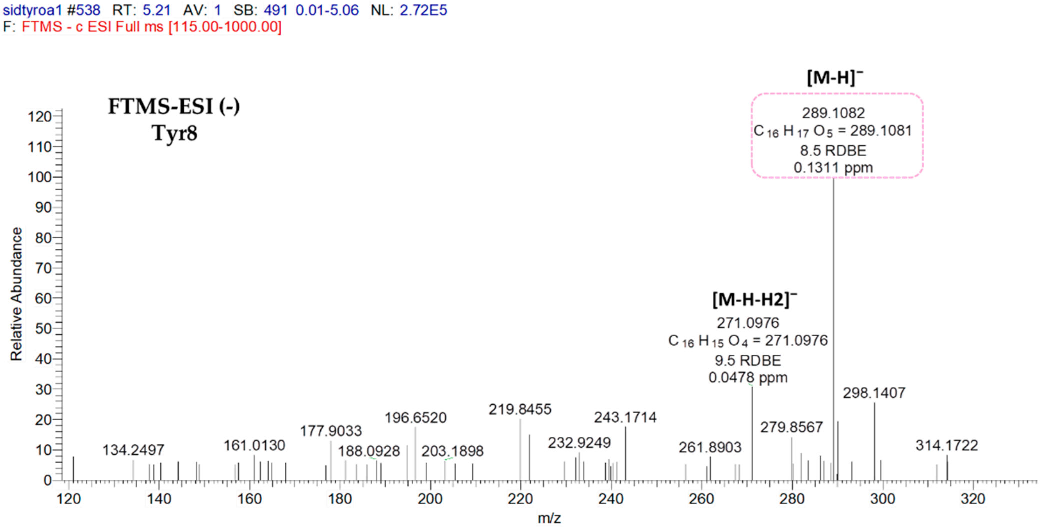

Figure S26: HRMS full scan spectrum of **Tyr8**.

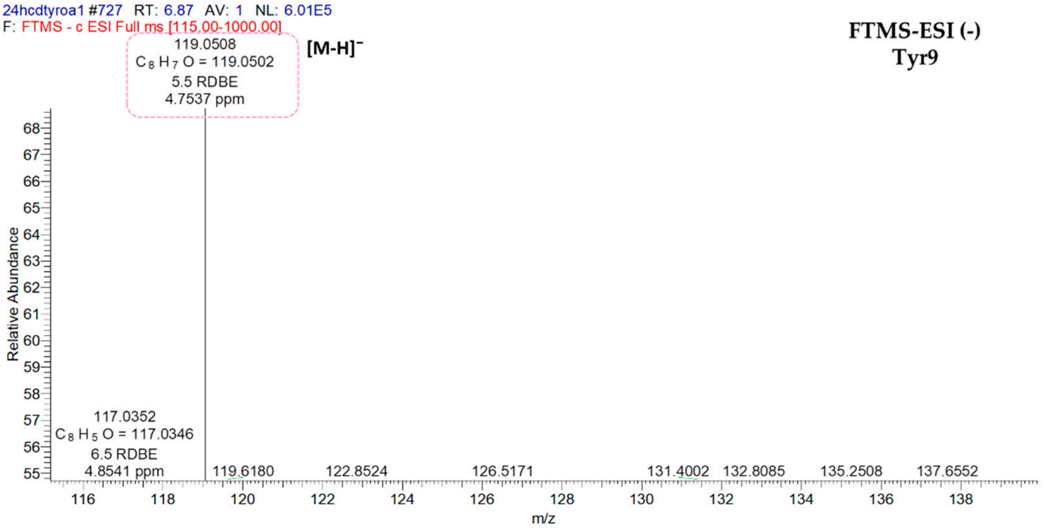

Figure S27: HRMS full scan spectrum of Tyr9.

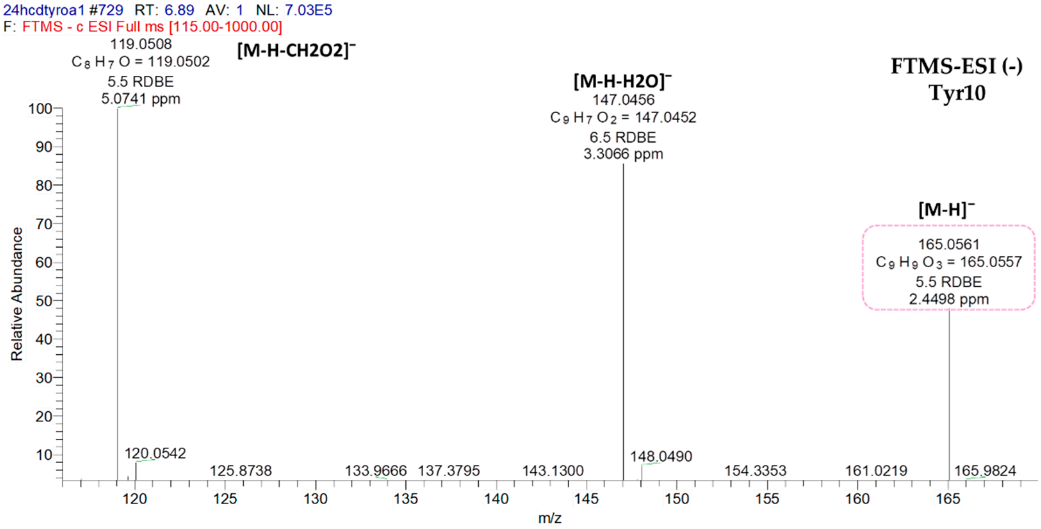

Figure S28: HRMS full scan spectrum of **Tyr10**.

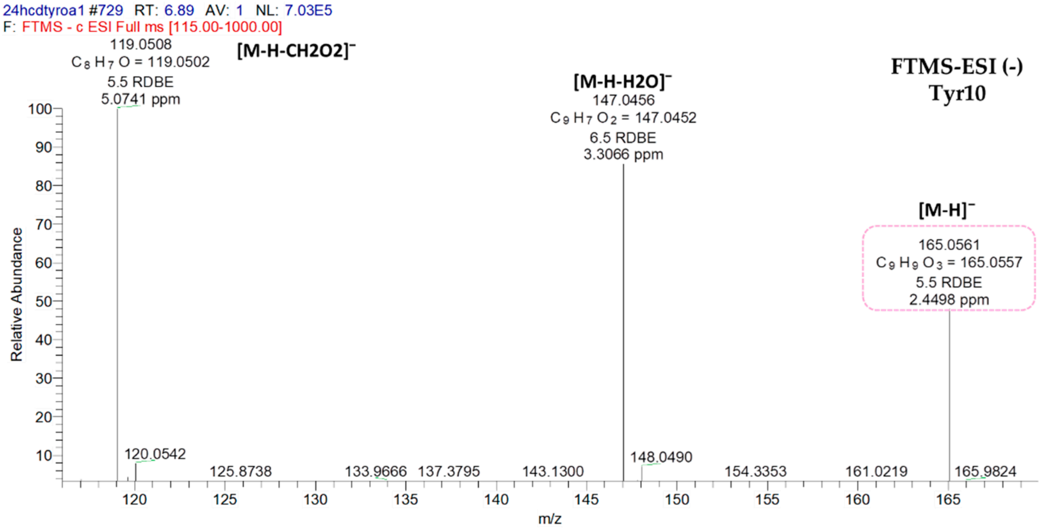

Figure S29: HRMS full scan spectrum of **Tyr12.**
